# Anchoring and Competition: Weakly Solvated Structure of Glymes Enhances Stability in Lithium Metal Batteries Operating under Extreme Conditions

**DOI:** 10.1002/anie.202511336

**Published:** 2025-08-14

**Authors:** Tianle Zheng, Mengqi Wu, Jianwei Xiong, Ming Yang, Wenzhe Guo, Qihan Zeng, Hongwei Yu, Tonghui Xu, Weiping Xie, Yiyao Xiao, Zhuijun Xu, Yuxin Liang, Zerui Li, Ruoxuan Qi, Guangjiu Pan, Xiaotang Shi, Hongbin Zhao, Xiaohong Li, Yongyao Xia, Ya‐Jun Cheng, Yonggao Xia, Peter Müller‐Buschbaum

**Affiliations:** ^1^ Chair for Functional Materials, Department of Physics, TUM School of Natural Sciences Technical University of Munich James‐Franck‐Str. 1 85748 Garching Germany; ^2^ Ningbo Institute of Materials Technology and Engineering Chinese Academy of Sciences 1219 Zhongguan West Rd Ningbo Zhejiang Province 315201 P.R. China; ^3^ Department of Chemistry College of Sciences Shanghai University Shanghai 200444 P.R. China; ^4^ College of Materials Science and Technology Nanjing University of Aeronautics and Astronautics Nanjing 210016 P.R. China; ^5^ College of Renewable Energy Hohai University 1915 Hohai Ave, Jintan District Changzhou Jiangsu Province 213220 P.R. China; ^6^ Center of Materials Science and Optoelectronics Engineering University of Chinese Academy of Sciences 19A Yuquan Rd, Shijingshan District Beijing 100049 P.R. China; ^7^ Department of Chemistry, Institute of New Energy Fudan University Shanghai 200433 P.R. China; ^8^ College of Chemistry Chemical Engineering and Materials Science Soochow University Suzhou Jiangsu 215123 P.R. China; ^9^ School of Materials Science and Chemical Engineering Ningbo University Ningbo 315211 P.R. China

**Keywords:** Lithium metal batteries, Lithium nitrate, Solvation structure, Weakly solvated structure, Wide temperature

## Abstract

Lithium metal batteries (LMBs) face challenges from unstable and fragile solid electrolyte interphases (SEIs). In this work, we successfully develop a novel electrolyte by effectively modulating the competitive solvation process in LMBs. In this formulation, the C─O─C motifs of glymes are competitively substituted by other anions and solvents to achieve single oxygen site coordination, thereby facilitating a weak solvation effect. At an apparent concentration of 1.25 M, a solvated sheath enriched with anions and single oxygen‐bound complexes is formed, which significantly enhances lithium metal compatibility and promotes rapid desolvation kinetics. The designed electrolyte using weakly solvated structures exhibits remarkable stability at both 25° and 80 °C, enabling the lithium iron phosphate (LFP)||Li cell to achieve over 2000 cycles (capacity retention: 90%) and 500 cycles (capacity retention: 96%), respectively. Interestingly, the low N/P ratio LFP||Li (N/P = 1.8) full battery maintains a stable capacity over 50 cycles, and the commercial 1.1 Ah LFP||Li pouch cell shows a great stability (capacity retention: 91.0%, CE: 99.82%) over 20 cycles. The distinctive solvation regulation strategy has paved a novel research avenue for the realization of high‐performance LMBs.

## Graphical abstract

Originally, lithium ions coordinate with multiple O atoms on diglyme, which would result in a strong binding energy. With the addition of LiNO_3_, the strong binding energy of NO_3_^−^ anchors the solvation structure on one side, and by adding a co‐solvent, diglymes with single O atom coordination are generated, which significantly reduces the desolvation energy of the solvation structure to improve the performance of Li metal batteries.

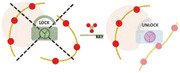

## Introduction

The ambitious pursuit of carbon neutrality has been propelling the advancement of high‐energy batteries, particularly in the area of lithium metal batteries (LMBs).^[^ ^1^, ^2^, ^3^, ^4^ ^]^ However, the devices still face numerous challenges, such as lower‐than‐expected energy density, lower safety, and a narrower working temperature range.^[^ ^5^ ^]^ The widely known issues primarily arise from unstable and fragile solid electrolyte interphases (SEIs), which exacerbate irreversible lithium loss and ultimately reduce the cycle life of the batteries, potentially leading to short circuits and thermal runaway.

Consequently, electrolyte engineering is considered one of the most promising and universally applicable strategies for mitigating side reactions between the lithium metal anode (LMA) and the electrolyte.^[^ ^6^, ^7^ ^]^ By modifying the composition of the electrolyte, it is possible to optimize both the structure and the chemical composition of the SEI, thereby enhancing cycling stability and the operation temperature window in LMBs. Recent research has demonstrated that innovative systems such as high‐concentration electrolytes (HCE), localized high‐concentration electrolytes (LHCE), and weakly solvating electrolytes (WSE) exhibit exceptional Coulombic efficiencies. These benefits are attributed to a reduction in free solvent molecules coupled with an increase in ion clusters like contact ion pairs (CIPs) and aggregates (AGGs), resulting in an SEI enriched with inorganic components.^[^ ^8^, ^9^ ^]^ However, HCE and LHCE approaches are often prohibitively expensive for commercial applications.^[^ ^10^, ^11^, ^12^ ^]^ Concurrently, weakly solvating solvents with limited capacity for solvation exhibit an insufficient dissolution efficacy for certain lithium salts characterized by high donor numbers in WSEs, as well as a restricted temperature range.^[^ ^13^, ^14^, ^15^, ^16^ ^]^ Therefore, developing new electrolytes that are cost‐effective while maintaining compatibility with various lithium salts represents a crucial strategy for advancing applications in LMBs.

**Scheme 1:**
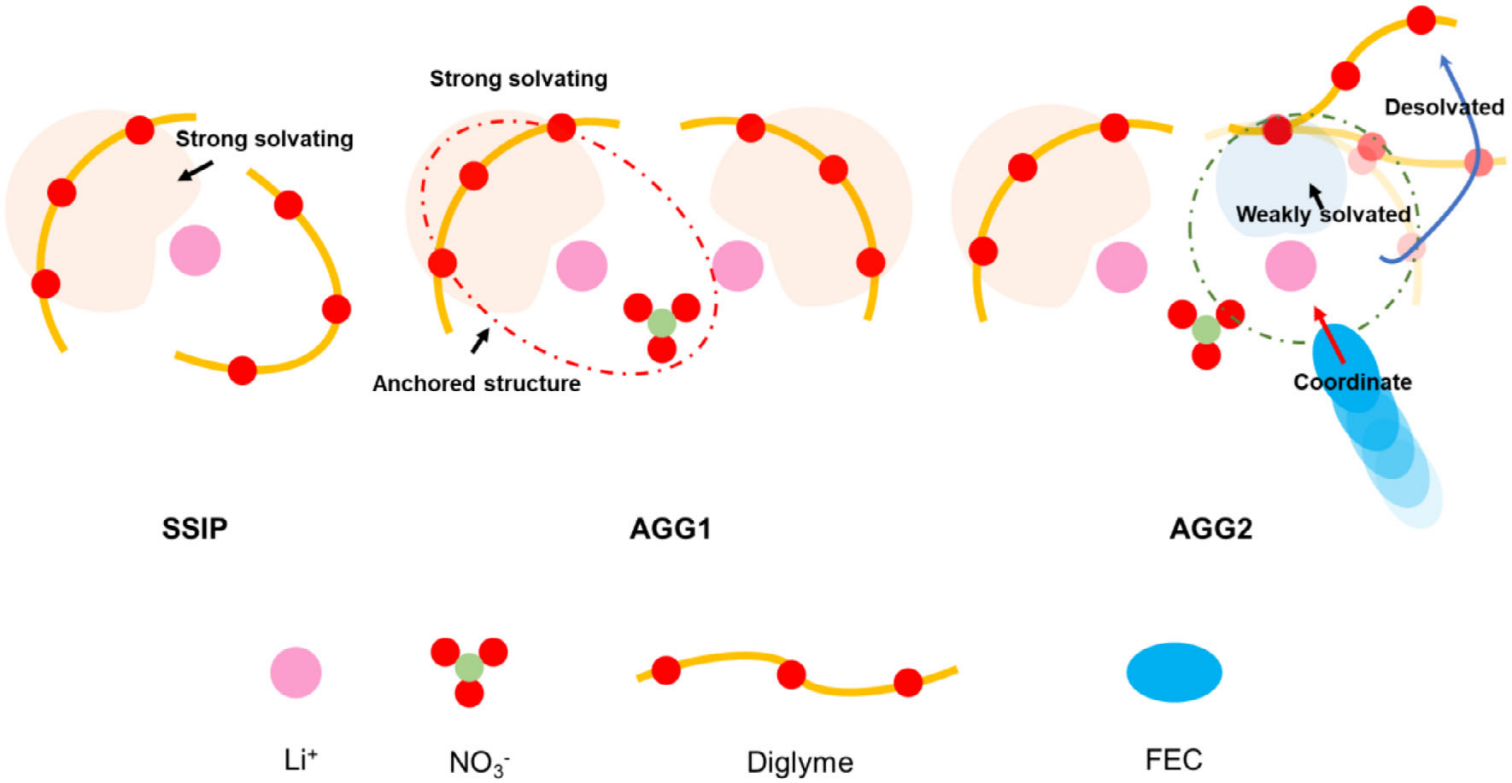
Evolution of competitive mediation to convert from strong solvating to weakly solvating.

To address these challenges, ether‐based solvents like diglyme (G2), triglyme (G3) and tetraglyme (G4) were initially selected as the base solvent due to their advantageous properties of low viscosity and high solubility for lithium salts, including LiNO_3_.^[^ ^17^, ^18^ ^]^ These solvents effectively dissolve a variety of lithium salts and demonstrate excellent compatibility with LMAs. Additionally, lithium bis(fluorosulfonyl)imide (LiFSI) was chosen as the primary salt owing to its high ionic conductivity and capacity to provide effective F sources for the SEI, rendering it an optimal choice.^[^ ^8^, ^19^, ^20^ ^]^ LiNO_3_ functions as a multifunctional additive that not only undergoes decomposition to generate products like Li_3_N, thereby facilitating the formation of a more stable SEI, but also exhibits robust interactions with Li^+^. Additionally, NO_3_^−^ possesses a symmetrical structure and exhibits a strong coordination ability with cations. Thus, it can serve as an “anchor” to stabilize specific regions of the solvated structure (Scheme 1), which approach has seldom been adopted in prior research. Finally, fluoroethylene carbonate (FEC) functions as a pseudo‐diluent and co‐solvent that complements the ether solvents and demonstrates notable differences in the solubility of LiFSI and LiNO_3_: while FEC effectively dissolves LiFSI, its solubility for LiNO_3_ is comparatively low. Therefore, it can be regarded as a pseudo‐diluent for LiNO_3_ to mediate the solvation structure.^[^ ^21^, ^22^ ^]^ Although LiNO_3_, FEC, and G2 have been widely used in recent studies, most of these works treat them merely as functional additives or common solvents, without thoroughly considering their intrinsic molecular structures or the unique solvation configurations they can form.^[^ ^23^, ^24^, ^25^ ^]^ In particular, the molecular interactions and coordination environments arising from their combination may give rise to distinct solvation structures, which could fundamentally impact the ion transport and interfacial stability. A deeper understanding of these effects is essential for rational electrolyte design beyond conventional additive‐based strategies.

In this study, we present a novel strategy to convert the multi‐O coordinate sites of G2 into weakly solvated sites through competitive solvation. Considering the exclusive ability of G2 to effectively dissolve lithium nitrate in the electrolyte, it plays a crucial role in anchoring a portion of the solvated structure. Simultaneously, G2 exhibits a remarkable capacity for attracting multiple Li ions. Now, the co‐solvent FEC will competitively occupy the remaining coordination sites on G2, compelling it to adopt a single O coordinate site and thereby manifesting as a form of weak solvation. (Scheme 1) This mechanism reduces the overall desolvation energy and significantly enhances the kinetic performance of the electrolyte. Furthermore, the solvated structure comprising FEC, NO_3_^−^, and FSI^−^ increases the inorganic component content, thereby facilitating the formation of a more stable SEI to improve the battery cycling stability.

## Results and Discussion

### Solvation Structure of the Electrolytes

**Figure 1:**
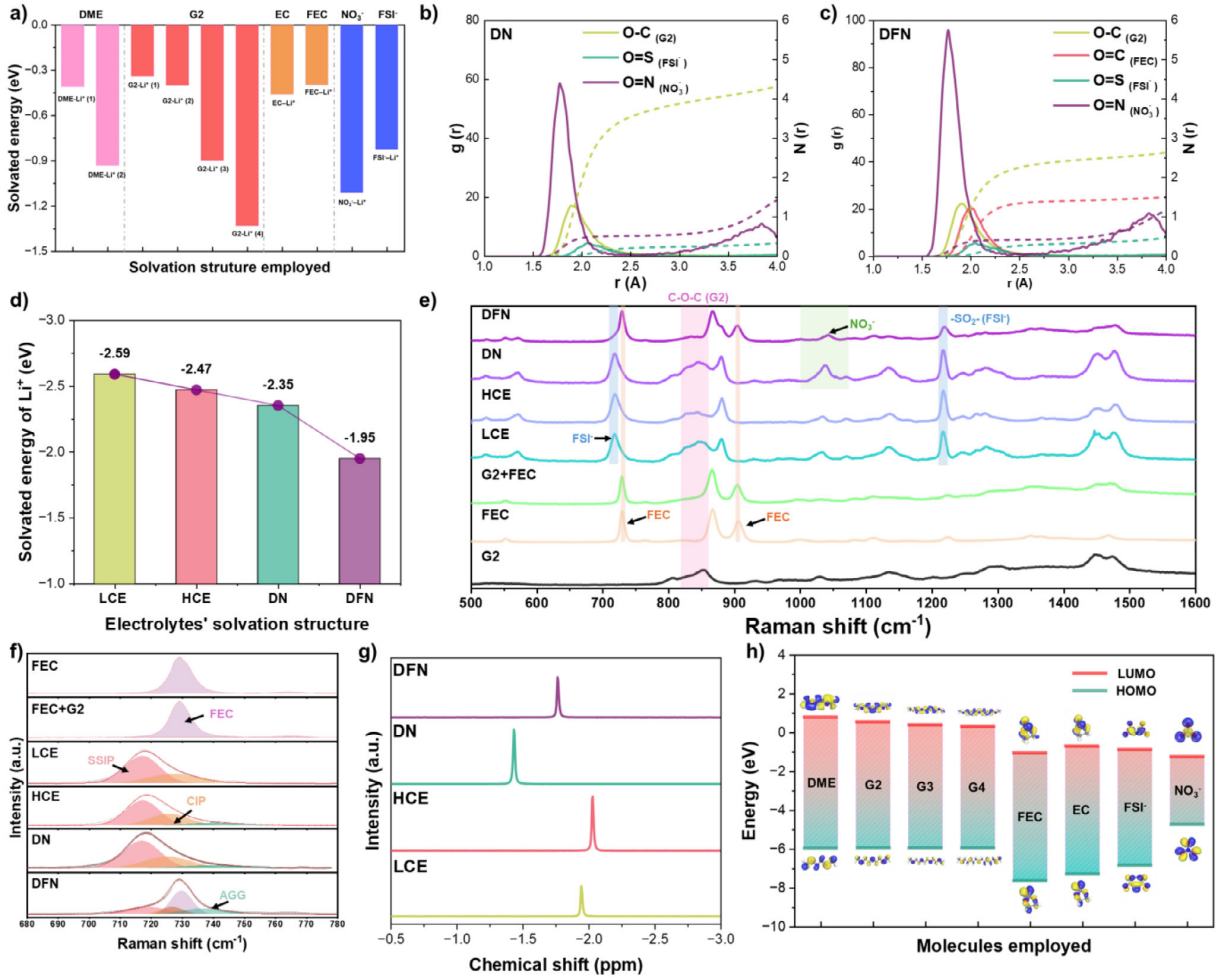
Solvation structures in electrolytes. a) Solvated energy of different adsorption structures. b), c) RDF results of DN and DFN. d) Desolvation energy per Li^+^ in various electrolytes. e), f) Raman spectra of different concentrations of electrolytes and solvents. g) ^7^Li NMR of various concentrations of electrolytes. h) LUMO/HOMO energy levels of different solvents and Li salts.

To gain insights into the solvation structures in diverse electrolytes, we have used density functional theory (DFT) calculations to elucidate the energy evolution of various locations or molecules for Li^+^ adsorption with chemically stable solvents and lithium salts. (Figure 1a and Table S1). Previous studies have demonstrated that the interaction between weakly solvated solvent molecules and Li^+^ is weak (dioxolane (DOL), tetrahydrofuran (THF), diethyl ether (DEE)), thereby reducing the desolvation energy of Li^+^ and consequently lowering the interfacial impedance of the electrolyte and the polarization voltage of the battery.^[^ ^26^, ^27^, ^28^ ^]^ Otherwise, linear glymes, like dimethoxyethane (DME) and G2, typically coordinate with Li^+^ using two or more O atoms, thereby generating a strong solvation effect, which enables them to dissolve salts with a strong binding energy like LiNO_3_, which was proved by the interaction energies of DME‐Li^+^ (2), G2‐Li^+^ (3) and G2‐Li^+^ (4). Among them, G2‐Li^+^ (4) has the strongest interaction due to the coordination of Li^+^ by three O atoms. Therefore, the ability of G2 to dissolve LiNO_3_ is stronger than that of DME. Interestingly, when linear glymes have only one O atom complexed with a Li^+^ (DME‐Li^+^ (1), G2‐Li^+^ (1), G2‐Li^+^ (2)), their relative interaction is weaker than that of weak solvating solvents. This behavior indicates that when such a configuration exists in the solvation shell, the solvation effect will be significantly reduced. Therefore, based on the above‐mentioned characteristics of binding energy and molecular structures, we designed four types of electrolytes to verify these insights: LCE (1.0 M LiFSI in G2), HCE (2.0 M LiFSI in G2), DN (1.0 M LiFSI and 0.5 M LiNO_3_ in G2), and DFN (1.0 M LiFSI/0.25 M LiNO_3_ in G2/FEC, *v/v* = 1:1).

Molecular dynamics (MD) simulation is further used to investigate the solvation structure of Li^+^ in different electrolytes. The solvent and anion coordination numbers (CN) obtained from analyzing radial distribution functions (RDF) are shown in Figures 1b,c, S1, and S2. Among the electrolytes, ether CNs decline in the order of LCE (4.15) > HCE (3.55) > DN (3.54) > DFN (2.26), indicating that the increase in lithium salt concentration and the addition of LiNO_3_ and FEC reduce the solvation effect of G2 and Li^+^. Simultaneously, in comparison to DN and DFN, the amount of NO_3_^−^ surrounding Li^+^ remains similar, whereas the number of FSI^−^ exhibits a notable increase. This observation further substantiates the viability of using FEC as a pseudo‐diluent for LiNO_3_ and suggests that incorporating FEC concurrently enhances the proportion of CIP.^[^ ^22^ ^]^ Figures S3–S6 show the structure of different electrolytes and the typical solvation structure in the corresponding electrolyte. The results also correspond to the RDF results. As the concentration of LiFSI increases, FSI^−^ is observed to be present in the solvation sheath. Upon addition of LiNO_3_ to the LCE, owing to the strong electrostatic force of NO_3_^−^, it can effectively bridge multiple lithium ions and connect various solvated structures. In DFN, the addition of FEC induces a robust electrostatic interaction between FEC and Li^+^, which enables FEC to effectively compete with O atoms in G2, thereby occupying the position typically held by ether‐O. Consequently, a high number of G2 molecules in DFN manifests a state with Li^+^ ions being coordinated with individual oxygen atoms, which causes a high diffusion coefficient as shown in Figure S7. Then, we combine the results of RDF to construct the typical solvation structures of corresponding electrolytes and further calculate the average solvation energy of per Li^+^. The increase in the groups of CIPs/AGGs can effectively reduce the desolvation energy (Figure 1b). Additionally, the co‐addition of FEC and LiNO_3_ leads to the formation of a distinctive solvation structure, which contains weakly‐solvated G2, resulting in a significant reduction in the average desolvation energy per Li^+^ in DFN to 1.95 eV (Figure 1d). Consequently, this enhancement effectively improves the kinetic performance of the electrolyte.

The Raman spectra of the relative solvents and electrolytes are shown in Figures 1e,f, S8, and S9. The free G2 molecules exhibit a prominent peak at 860 cm^−1^, which corresponds to the stretching modes of C─O─C.^[^ ^29^ ^]^ Upon addition of LiFSI or LiFSI/LiNO_3_ to G2, a distinct peak at 870 cm^−1^ emerges, indicating the presence of Li‐coordinated G2 groups. The optical signatures of free G2 solvents are significantly attenuated, particularly in the DFN electrolyte. This observation is further corroborated by the Fourier Transform Infrared (FTIR) spectra (Figure S10), indicating a complete coordination between the G2 solvent and Li^+^.^[^ ^30^ ^]^ Additionally, the peaks corresponding to the S─N stretching vibration of FSI^−^ (680–780 cm^−1^) and NO_3_^−^ vibration (1000–1075 cm^−1^) exhibit high sensitivity toward variations in Li coordination (Figures 1e and S8).^[^ ^31^, ^32^ ^]^ In LCE, there are 76.4% free FSI^−^ (at 720 cm^−1^), 23.6% contact ion pairs ([CIPs], at 728 cm^−1^), and barely visible aggregates ([AGGs], at 740 cm^−1^). In HCE, a higher content of CIPs and AGGs is observed, indicating a change in the solvation structure. Upon the addition of 0.5 M LiNO_3_, this ratio of CIPs/AGGs further increases to 40.1%, suggesting an increased presence of inorganic components within the DN electrolyte's solvation structure. Moreover, in the DFN electrolyte, the proportion of CIPs/AGGs content reaches 57.1%, while the peak corresponding to NO_3_^−^ in CIPs/AGGs shifts to approximately 1045 cm^−1^ because the FEC occupies the original site of G2, thereby leading to more G2 molecules contacting with LiNO_3_ by a single ether‐O atom. The above results significantly confirm that the introduction of LiNO_3_ and FEC can further increase the solvated G2 and CIPs/AGGs, enhancing the ion‐dipole interactions in the solvation structure, which aligns with the results of MD simulations.

The nuclear magnetic resonance (NMR) signals ^1^H and ^7^Li in the electrolytes help to further analyze the solvation structure in various electrolytes. (Figures 1g, S11, and S12; Table S2 and S3) ^7^Li NMR spectra display an upfield shift as the concentration of LiFSI increases, suggesting an increased electron density around Li^+^ due to a stronger bond energy between Li^+^ and anions.^[^ ^33^ ^]^ In contrast, the effects of LiNO_3_ on the local environment of Li in the electrolytes show a more upfield shift, indicating that the electron density around Li decreases with the gradual increase in the number of LiNO_3_, which is consistent with previous reports.^[^ ^34^, ^35^ ^]^ With the addition of FEC, the signal moves toward upfield due to FEC's weaker electron‐accepting ability compared to NO_3_^−^, but the electron density around Li remains lower than that of LCE.^[^ ^20^ ^]^ The structure evolution of G2 in different electrolytes is further confirmed by ^1^H NMR. The characteristic peaks corresponding to the H atoms located on different sides (A, B, and C as shown in Figure S12) exhibit a shift toward higher fields with increasing concentration of the lithium salt, indicating an increase in the electron density. This shift corresponds to the number of G2 molecules coordinating Li^+^. In the DFN electrolyte with an apparent concentration of only 1.25 M, the electron density is found to be maximum compared with the other electrolytes, and the distance between peaks A and C is also observed to be the largest.(Tables S2 and S3) These observations suggest that more Li^+^ ions are coordinated with O atoms in the CH_3_OCH_2_ groups at the molecular edge, resulting in a significant difference in the electron density between peaks A and C corresponding to CH_3_O─ and ─CH_2_O─. This finding further supports the formation of a structure in G2, where Li^+^ ions are coordinated by a single oxygen atom, deriving from the competitive solvation.

Furthermore, Figures 1h and S13 also show the energy levels of the molecular orbits of different solvents, anions, and ion pairs. Obviously, DME, G2, G3, and G4 exhibit decreasing energy levels of the lowest unoccupied molecular orbital (LUMO) with the increasing number of ─CH_2_─O─CH_2_─ units, indicating an enhanced tendency to undergo reduction. However, these solvents have similar and high energy levels of the highest occupied molecular orbital (HOMO), indicating that they are prone to be oxidized easily at the cathode. To obtain a more stable electrolyte, we compare several conventional solvents and lithium salts. Both FEC and NO_3_^−^ exhibit similar and significantly lower LUMO levels, indicating their potential for reacting to similar potentials and forming stable SEI films. Moreover, FEC demonstrates a lower HOMO level, which can effectively enhance the oxidation resistance of the electrolyte and compensate for the limitations associated with ether‐based electrolytes.

### Electrochemical Performance of the Electrolytes

The 4 kinds of electrolytes are used to assemble Li||Cu half‐cells and Li||Li symmetric cells to investigate their electrochemical performance. The high‐voltage tolerance of electrolytes is assessed by conducting linear sweep voltammetry (LSV) measurements on the Al collector (Figure S14). Obviously, commercial electrolyte, LCE, and HCE begin to experience a gradual increase in the current below 4.0 V. HCE is more prone to oxidation than LCE, which means that this electrolyte is more likely to cause corrosion of aluminum in high‐voltage environments, which can be attributed to the intrinsic oxidative instability of G2 and the greater amount of LiFSI in HCE.^[^ ^36^ ^]^ In contrast, with the introduction of LiNO_3_, the DN electrolyte demonstrates a better antioxidant capacity, enabling the voltage window of the ether‐based electrolyte to exceed 4.6 V. The incorporation of FEC can slightly elevate the voltage window and decrease the corrosion current.

**Figure 2:**
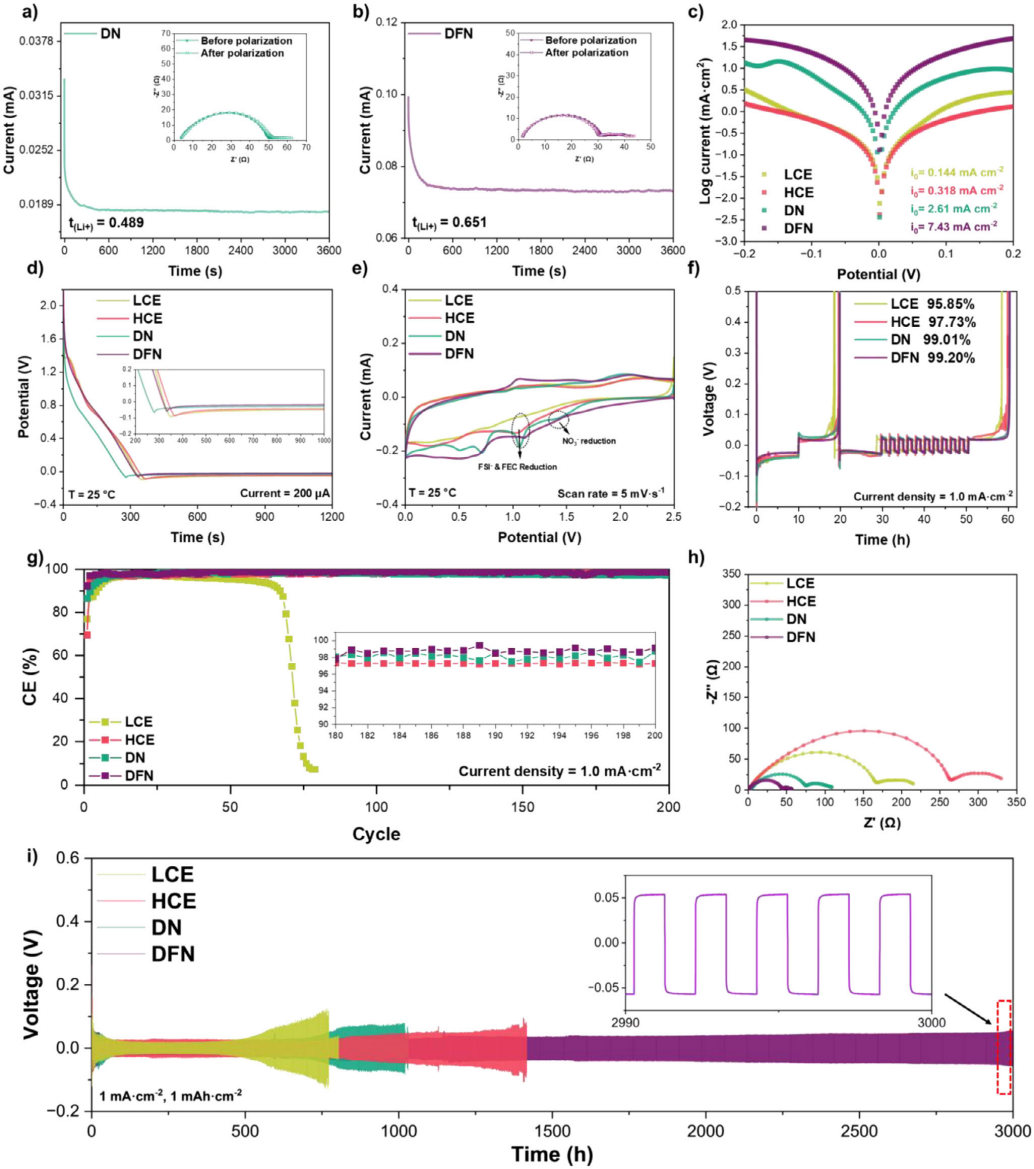
Li plating/stripping performance in various electrolytes. a), b) Li^+^ transference number of DN and DFN. c) Tafel plots of Li plating/stripping. d) Discharge curves with current density at 200 µA. e) CV curves of 0–2.5 V at a scan rate of 5 mV s^−1^. f) Li plating/stripping curves at a current density of 1 mA cm^−2^ and capacity of 1 mAh cm^−2^. g) Long‐term cycling of Li||Cu half cells at a current density of 1 mA cm^−2^ and capacity of 1 mAh cm^−2^. h) Nyquist plots of electrochemical impedance spectra for Li||Li symmetric cells after 8 h of rest. i) Long‐term cycling of Li||Li symmetric cells at a current density of 1 mA cm^−2^ and capacity of 1 mAh cm^−2^.

The Li transference number of electrolytes is a crucial property that plays an important role in enhancing the performance of LMBs.^[^ ^22^, ^37^ ^]^Therefore, Li||Li symmetric cells are used to evaluate this parameter derived by the Bruce–Vincent method, and the results obtained at room temperature are presented in Figures 2a,b and S15.^[^ ^38^ ^]^ Among all the electrolytes, DFN possesses the highest Li transference number, attaining 0.65. Due to a considerable number of CIP/AGG structures in the solvation structure of DFN, it leads to the presence of multiple Li ions in the solvation structure, which is highly advantageous for enhancing the cycling stability of the battery and reducing polarization. In contrast, DN and HCE likewise possess a considerable number of CIP/AGG structures, thus also demonstrating a higher transference number than LCE. Moreover, DFN also has the largest exchange current densities (*i_0_*) calculated from Tafel curves in Figure 2c, which also confirms its excellent ionic kinetics and reversible Li plating/stripping compared with other electrolytes.^[^ ^28^, ^39^, ^40^ ^]^

The Li deposition kinetics are further investigated in a Li||Cu half‐cell using CV within the potential range of −0.3 to 0.6 V. (Figure S16) The ranking of current density from large to small is DFN, DN, HCE, and LCE. HCE and LCE exhibit poor reaction kinetics, which is attributed to their larger desolvation energy. While both DFN and DN have much higher Li plating/stripping currents, further confirming that the addition of LiNO_3_ and FEC reduces the desolvation energy of the electrolyte. Additionally, the nucleation onset potential in the DFN electrolyte exhibits a reduction of 13, 44, and 57 mV compared to DN, HCE, and LCE, respectively, thereby providing further confirmation of the enhanced reaction kinetics for lithium deposition in DFN. To further investigate the deposition mechanism, both CV and chronopotentiometry tests are done to gain a comprehensive understanding of the formation of SEI film and the electrochemical reduction process. Figure 2d shows the process of SEI formation at a low current density of 200 µA. The reduction process of LCE and HCE initiates at approximately 0.8 V, which corresponds to the decomposition of the lithium salt. Notably, the reduction process of HCE exhibits a prolonged duration compared to that of LCE due to the higher concentration of LiFSI. In contrast, DFN demonstrates a distinct film formation process and the lowest overpotential, which implies its outstanding kinetics. The CV curves of several electrolytes in the voltage range of 0–2.5 V are presented in Figures 2e and S17, where Li deposition is prevented while allowing for electrolyte decomposition. The reduction peak around 0.5 V is attributed to the underpotential deposition of Li.^[^ ^41^ ^]^ The reduction peak around 1.1 V can be attributed to the reaction of FSI^−^, which rises with an increase in concentration.^[^ ^42^, ^43^ ^]^ DN shows an additional reduction peak located around 1.6 V, which represents the decomposition of LiNO_3_.^[^ ^31^, ^44^, ^45^ ^]^ In contrast, DFN shows a lower FEC and FSI^−^ peak, indicating inhibited decomposition of the electrolytes.^[^ ^42^ ^]^

The cycling stability of Li anodes is highly dependent on the Coulombic efficiency (CE) and the utilization of Li in each cycle. In practical LMBs, it is common for a portion of Li metal to remain on the anode, as a complete removal from the current collector is not achieved.^[^ ^46^ ^]^ Figure 2f presents the results of the Aurbach protocol applied in Li||Cu half‐cells using various electrolytes for CE calculation.^[^ ^47^ ^]^ The DFN electrolyte exhibits a remarkable Li plating/stripping CE of 99.20%, surpassing the values obtained in LCE (95.85%) and HCE (97.73%). Moreover, as depicted in Figure 2g, the CE of the cells using LCE exhibits a rapid decay below 90% within less than 75 cycles. Conversely, the HCE, DN, and DFN electrolytes demonstrate a significantly enhanced cycling stability. Specifically, during the initial 200 cycles, the average Li CE achieved by DFN surpasses that of HCE (97.91%) and DN (98.13%), reaching approximately 98.6%. Figure 2h shows the impedance of Li||Li symmetric cells before working. HCE demonstrates the highest interfacial impedance, which is ascribed to the overly high lithium salt concentration, resulting in an excessively high electrolyte viscosity. Upon the addition of LiNO_3_, the impedance of DN decreases significantly compared with that of LCE, while DFN possesses the smallest interfacial impedance, which is also in accordance with the previous kinetic tests and MD simulation results. Moreover, the DFN electrolyte exhibits outstanding compatibility and exceptional stability in Li||Li symmetric cells and reliability during calendar aging. (Figure 2i) The overpotential of cells with LCE exhibits a significant increase after 500 h, primarily attributed to the pronounced decomposition of electrolyte and accumulation of the SEI layer. When the concentration increases, the cell exhibits a cycling life of 1400 h. Additionally, the addition of LiNO_3_ also improves the cycling life due to the inorganic products decomposed by LiNO_3_. In contrast, the DFN electrolytes can support that the cells work more than 3000 h with a very low polarization due to the high Li^+^ transference number and small interfacial impedance. The CV of Li||Li symmetric cells also confirms this finding (Figure S18). The electrochemical impedance spectroscopy (EIS) results of various cell cycles demonstrate a significant decrease in battery impedance following the charge–discharge process, and the impedance values are observed as follows: DFN < DN < HCE < LCE, which is attributed to the formation of SEI films on the electrode surfaces (Figure S19). Notably, the impedance value of DFN reaches a relatively small value in the 20^th^ cycle, while the other electrolytes still need many cycles of reaction. This behavior further indicates the outstanding lithium metal compatibility of the DFN electrolyte.

### Performance of Li Metal Anode

**Figure 3:**
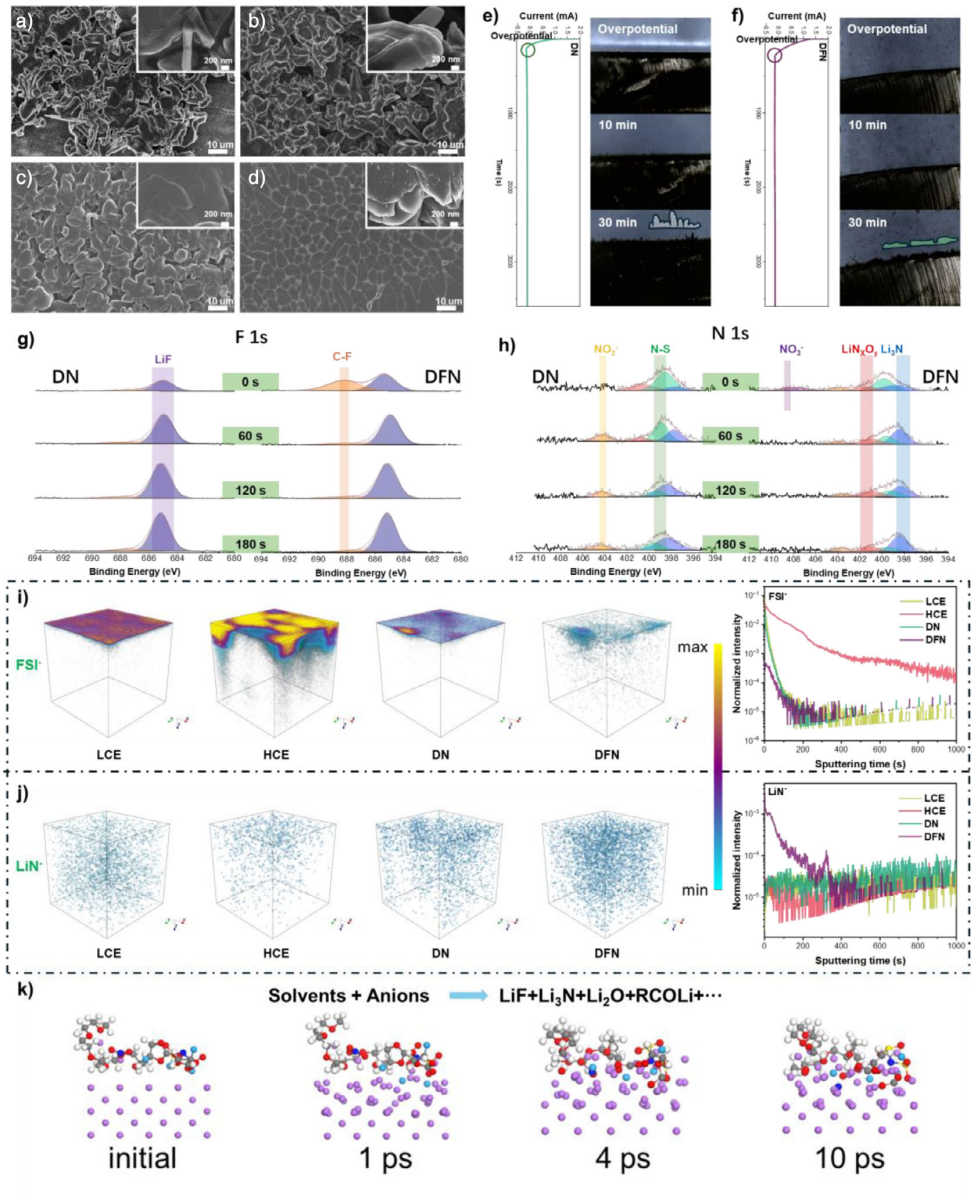
Morphology, in situ electrochemical deposition, and chemical compositions of SEI. SEM images for the surface of Li electrodeposits after the first plating on the Cu foil at 0.5 mA cm^−2^ and 3 mAh cm^−2^ in a) LCE, b) HCE, c) DN, and d) DFN. In situ electrochemical optical‐microscope images of e) DN and f) DFN. XPS spectra of g) F 1s and h) N 1s of the Li deposition of Ar^+^ sputtering in DN and DFN. i), j) 3D reconstruction for the sputtered volume and composition distribution of a 30‐cycled Li metal anode with different electrolytes: (i) FSI^−^ and (j) LiN^−^. k) Evolution of Li metal interface and solvation structure results from AIMD.

To investigate the compatibility with LMA further, Li metal deposition is performed on a copper foil at a plating density of 3 mAh cm^−2^ using various electrolytes. Scanning electron microscopy (SEM) is applied to observe morphology. (Figure 3a–d) The presence of dendrites called “dead Li” in both LCE and HCE deposits can be observed, which potentially enhances the contact area with the electrolyte and accelerates side reactions. In DN and DFN electrolytes, the deposition stabilities are both significantly improved. In DN electrolyte, the surface shows many flat and big Li metal structures, indicating that the addition of LiNO_3_ changes the shape of the Li deposition, like the previous research showed.^[^ ^31^, ^42^, ^48^, ^49^, ^50^ ^]^ In contrast, DFN exhibits Li metal sheets that are remarkably flat, dense, and uniform with minimal boundaries. The flat and dense structure of this shape is highly conducive to the utilization of lithium‐metal, as it effectively minimizes side reactions and prevents the formation of detrimental lithium dendrites. These uniform structures provide compelling evidence for the exceptional stability and efficiency achieved in cycling lithium metal electrolytes.^[^ ^51^ ^]^ The cross‐sectional profile depicted in Figure S20 also illustrates the thickness of Li deposition: DFN (14.44 µm) < DN (16.09 µm) < HCE (25.86 µm) < LCE (28.40 µm). This observation confirms that DFN exhibits a superior compatibility for generating dense and uniform deposition.

In order to gain further insights into the evolutionary process of Li plating, we use an in situ electrochemical optical‐microscope technique across four different electrolytes with varying current densities, as depicted in Figures 3e,f, S21, and S22. The Li plating in HCE and LCE electrolytes exhibits the formation of fluffy and uneven structures accompanied by dendrites. Although, in the case of DN, the sizes are larger compared to that observed in LCE and HCE, it still lacks uniformity. In sharp contrast, the DFN electrolyte exhibits a more uniform, flatter, and larger bulk deposition than the previous observation. This finding is consistent with the SEM characterizations and demonstrates excellent compatibility with the Li metal anode.

The chemical composition and structure of the SEI layer formed by different electrolytes are analyzed using X‐ray photoelectron spectroscopy (XPS), energy dispersive spectrometer (EDS), and time‐of‐flight secondary ion mass spectrometry (TOF‐SIMS). Based on the XPS depth profiles and corresponding F 1s, N 1s, O 1s, and C 1s patterns depicted in Figures 3g,h and S23–S27, it can be observed that the outer layers of the SEI primarily consist of organic species resulting from solvent and anion decomposition, while inorganic species are predominantly present in the inner layers. In LCE and HCE, the distribution of various products is approximately similar. The primary distinction lies in the augmentation of the peaks at 399 eV (FSI^−^) and 688 eV (S‐F), resulting from a substantial amount of FSI^−^ decomposition.^[^ ^51^ ^]^ The S element is exclusively derived from the decomposition of FSI^−^, thus indicating an intensified FSI^−^ decomposition in HCE, which consequently leads to a significant augmentation in the concentration of the F element. In DN, the decomposition of anions is mitigated, which is ascribed to the fact that LiNO_3_ substitutes for a portion of FSI to undergo a preferential reaction in the solvation sheath. Simultaneously, due to the reduction reaction resulting from the addition of LiNO_3_, new peaks emerge at 404 and 401 eV, representing the generation of LiNO_2_ and LiNxOy.^[^ ^51^ ^]^ In contrast, the involvement of FEC in the DFN electrolyte complicates the reaction. First, due to its pseudo‐diluent role, FEC replaces a portion of G2 and NO_3_^−^ in the solvation sheath, leading to the formation of C─F bonds in the outermost SEI, which is attributed to the ring reaction of FEC. Second, by synergizing with LiNO_3_, FEC promotes the generation of a larger quantity of LiF (685 eV), Li_3_N (398 eV), Li_2_O (529 eV), and Li_2_CO_3_ (290 eV). Consequently, this facilitates the formation of a more stable and robust SEI film that enhances battery cycling stability. Additionally, the EDS chemical mapping also confirms the results of XPS in Figures S28–S31. The deposition of lithium on the surface using DFN exhibits the most homogeneous distribution of elements, with the highest contents of F and N elements, while the S element content is found to be the lowest. These findings provide evidence for effective inhibition of anion decomposition.

TOF‐SIMS provides a more detailed composition distribution of the SEI in different electrolytes. (Figures 3i,j and S32–S37) Evidently, HCE exhibits the most intense FSI^−^ signal and is predominantly localized on the surface, indicating a substantial decomposition of anions. This finding also explains the relatively high concentration of the F element in the outer layer of HCE; however, it displays significant non‐uniformity, thus impeding stable battery cycling. These findings are consistent with XPS analysis results. The addition of lithium nitrate in DN is found to effectively mitigate the decomposition of FSI and, to some extent, promote the presence of inorganic components within the SEI layer, including Li_2_O, Li_3_N, LiF, and Li_2_CO_3_. Furthermore, DFN demonstrates significantly elevated levels of LiN^−^ and LiO^−^ in comparison to other electrolytes, while concurrently exhibiting a tenfold reduction in S content. Moreover, it also shows the lowest G2 decomposition observed by CH_2_O^−^ content evolution. These observations imply that the incorporation of FEC and LiNO_3_ competitively interacts with anions, restraining their decomposition and resulting in the formation of a more stable SEI layer enriched with higher proportions of inorganic constituents.

To gain a deeper understanding of the impact exerted by its distinctive solvation sheath on the lithium deposition reaction, we use Ab initio molecular dynamics (AIMD). (Figures 3k, S38, and S39) In the initial stage of the reaction, two F atoms are released by the anion, resulting in the production of 2 LiF and LiNSO_4_ around 900 fs. The simultaneous occurrence of NO_3_^−^ and FEC solvent reactions is consistent with their respective LUMO energy levels. Due to the influence of the electric field, NO_3_^−^ enters the lithium metal and undergoes a gradual oxygen atom loss, with the transformation of NO_2_^−^ into NO^−^ being identified as the rate‐determining step. Eventually, one nitrate radical will decompose to yield 3 Li_2_O and one Li_3_N, while Li_3_N is found to be situated deeper within the SEI layer. The individual FEC will initially undergo a ring‐opening reaction, leading to the detachment of the F atom and subsequent formation of LiF, Li_2_O, and RCOLi. Alternatively, due to the adsorption of Li ions, preferential cleavage of the C─F bond in Li‐FEC occurs, distinguishing it from that observed in individual FEC. This ultimately results in the generation of LIF, RCOLi, and LiC_X_O_Y_. The analytical results of XPS and TOF‐SIMS further support this finding, reinforcing the notion that the combination of LiNO_3_ and FEC in DFN can synergistically induce the formation of a substantial number of inorganic components, thereby facilitating the development of a stable and homogeneous SEI film.

### Performance of LFP/Li Cells

**Figure 4:**
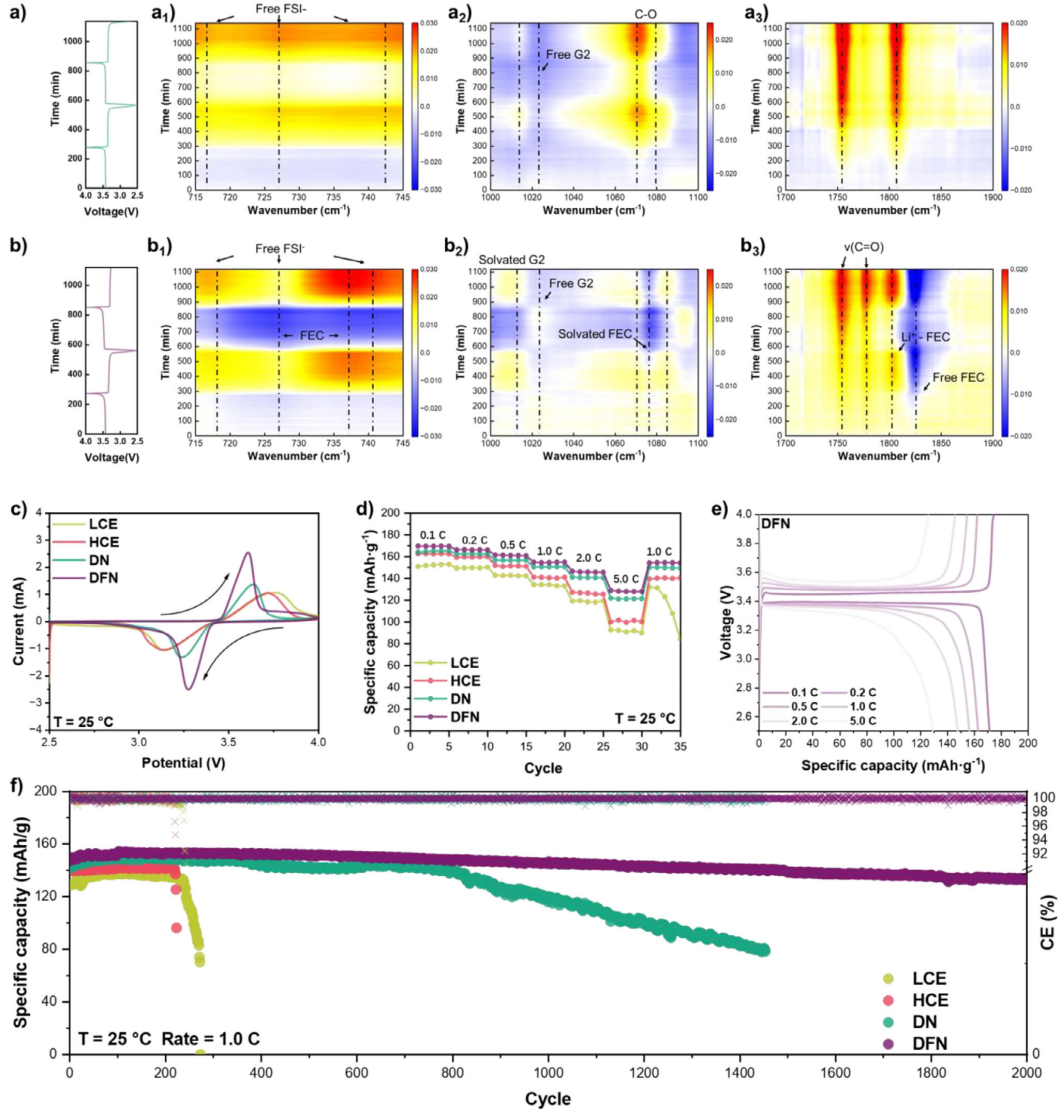
Voltage–time curves and corresponding contour map of the FTIR spectra during the charge/discharge process when using the a)–(a_3_) DN and b)–(b_3_) DFN. c) LFP CV curves of 2.5–4.0 V with scan rate at 0.1 mV s^−1^ in different electrolytes. d) Rate performance of LFP||Li half‐cells in different electrolytes. e) Charge–discharge curves of LFP||Li half‐cells at different rates in DFN electrolyte. f) Long‐term cycling of LFP||Li half‐cells at a rate of 1.0 C in different electrolytes.

The evolution of the solvation structure at the cathode‐electrolyte interface is investigated using in situ attenuated total reflection‐Fourier transform infrared (ATR‐FTIR) spectroscopy to establish correlations with different electrolytes. (Figures 4a,b and S40–S43) In LCE, during the first cycle of charging, once the positive electrode loses electrons, it will release Li ions, which leads to an increase in the concentration of Li ions at its interface. Simultaneously, a portion of the FSI anions will undergo oxidation and decomposition, thereby generating a negative signal in the 720–740 cm^−1^ regions. Upon battery discharge, the migration of Li ions from the electrolyte into lithium iron phosphate (LFP) leads to a decrease in the overall concentration of Li ions at the interface. Due to G2's superior ability to form complexes with Li ions compared to FSI anions, there will be a relative increase in the concentration of FSI. This behavior is evidenced by the intensified peak intensity of the G2 solvent at 1010 and 1070 cm^−1^. The peaks appearing at 1755 and 1810 cm^−1^ are attributed to *v*_(C_═_O)_, and the signal remains enhanced during the subsequent charging and discharging processes, which is attributed to the accumulation of reaction products. In the DN electrolyte, the signal in the 715–745 cm^−1^ region is consistently lower than that observed in LCE. (Figure 4a_1_) This observation suggests that the addition of LiNO_3_ has stabilized the solvation structure, thereby diminishing the competitive interactions between G2 and FSI. Furthermore, Figure 4a_2_ illustrates a progressive decrease in the intensity of solvated G2 relative to LCE with each discharge cycle. A comparative analysis of peaks at 1080, 1755, and 1810 cm^−1^ reveals that as LiNO_3_ concentration increases, there is an enhancement in products containing C═O while those containing C─O diminish correspondingly. (Figure 4a_2_,a_3_) Conversely, the DFN electrolyte exhibits a significant increase in peak complexity due to FEC incorporation; notably, the intensity of free FEC solvent diminishes throughout both charging and discharging cycles (1830 cm^−1^). The peak at 727 cm^−1^ associated with FEC overlaps with that of FSI^−^; thus, the relative intensity of FSI can be determined from its corresponding peak at 717 cm^−1^, which value is slightly lower than those observed for DN and LCE, indicating a more stable solvation environment. Additionally, reduced intensities are observed for the 1010 and 1070 cm^−1^ peaks, reflecting a diminished solvation capacity for G2 within DFN. This phenomenon can be attributed to competition from FEC replacing some fraction of G2, which is consistent with MD results. Regarding reaction products, DFN demonstrates fewer C─O species alongside an increased variety of C═O compounds conducive to enhanced formation rates of Li_2_CO_3_.

The CV curves of Li/LFP half‐cells at a scan rate of 0.1 mV s^−1^ at room temperature are presented in Figure 4c. The oxidation and reduction peaks of LFP in both LCE and HCE exhibit a broad and truncated shape, attributed to their limited kinetic properties. Following the addition of LiNO_3_, the separation between the oxidation and reduction peaks is reduced, accompanied by a slight increase in peak intensity. In contrast, DFN demonstrates both the highest peak intensity and the smallest inter‐peak distance, suggesting lowered polarization and improved charge‐transfer ability of the electrolyte, which can promote the rate capacity.^[^ ^52^ ^]^

The electrochemical performance of LFP||Li cells with various electrolytes at rates from 0.1 to 5.0 C is depicted in Figures 4d,e and S44. Consistent with the findings from CV, DFN demonstrates superior rate capability, achieving a specific capacity of 130 mAh g^−1^ at a current density of 5.0 C. In addition, the charge–discharge curves reveal that DFN possesses the lowest polarization voltage due to the excellent kinetics of DFN. However, due to the relatively poor stability of LCE, overcharging begins to occur when the battery is cycled back to 1.0 C. Besides, long‐term cycling performance of the cell is conducted with 1.0 C charge–discharge. The cells using HCE and LCE exhibit suboptimal performance and reduced stability (Figure 4f). The capacity exhibits a conspicuous decline, accompanied by an abrupt reduction in CE, at 220 and 240 cycles, ultimately leading to short‐circuiting (Figure S45). This behavior can be attributed to the narrower oxidation window of HCE and LCE, leading to intensified electrolyte consumption. DN demonstrates commendable stability, maintaining a capacity retention rate of 93.3% after 800 cycles. However, this is followed by a pronounced decline in capacity due to the depletion of the electrolyte and increasing polarization of cells. The cell with DFN, in stark contrast, exhibits exceptional cycling performance by achieving a capacity retention of 97.13% after 1000 cycles and 90.76% after 2000 cycles, while maintaining nearly identical voltage polarization as the initial state due to the formation of a robust cathode electrolyte interfacial layer (CEI layer). This behavior is consistent with findings, which demonstrated that a CEI formed by FEC and LiNO_3_ additives can efficiently stabilize ether‐based electrolytes at the cathode interface.^[^ ^53^, ^54^ ^]^ In Figure S46, the surfaces of the LCE and HCE electrodes fail to observe the complete LFP particles, suggesting that the oxidation of the electrolyte has impaired the electrode structure. By contrast, the battery incorporating DN and DFN electrodes can still preserve a complete LFP structure after 100 cycles.

XPS was further used to detect the components of the CEI for explaining the interface stability (Figures S47 and S48). Different from the SEI, the composition of the CEI on the cathode surface with different electrolytes exhibits relatively minor differences. Among them, DFN can increase the highest contents of LiF, LiN_X_O_Y_, and Li_3_N, which endows its CEI with a higher inorganic content and the best ionic conductivity. This protective CEI effectively suppresses solvent oxidation, even if the solvent itself exhibits lower oxidative stability.^[^ ^53^, ^54^ ^]^ The most significant disparity originates from the S element, representing the decomposition of FSI^−^. The content of the S element at the cathode interface is as follows: LCE > HCE > DN > DFN. In the battery using DFN, the products containing the S element are nearly non‐existent, indicating that the addition of LiNO_3_ and FEC jointly suppresses the decomposition of anions, thereby reducing the consumption of the electrolyte and promoting the cycling stability of the battery.

### Performance of LMBs under Extreme Conditions

To further investigate the performance of LMBs under extreme conditions, we use LFP||Li half cells to assess their stability at 80 °C and evaluate the stability of LFP||Li full cells with varying thicknesses of Li foils at room temperature.

**Figure 5:**
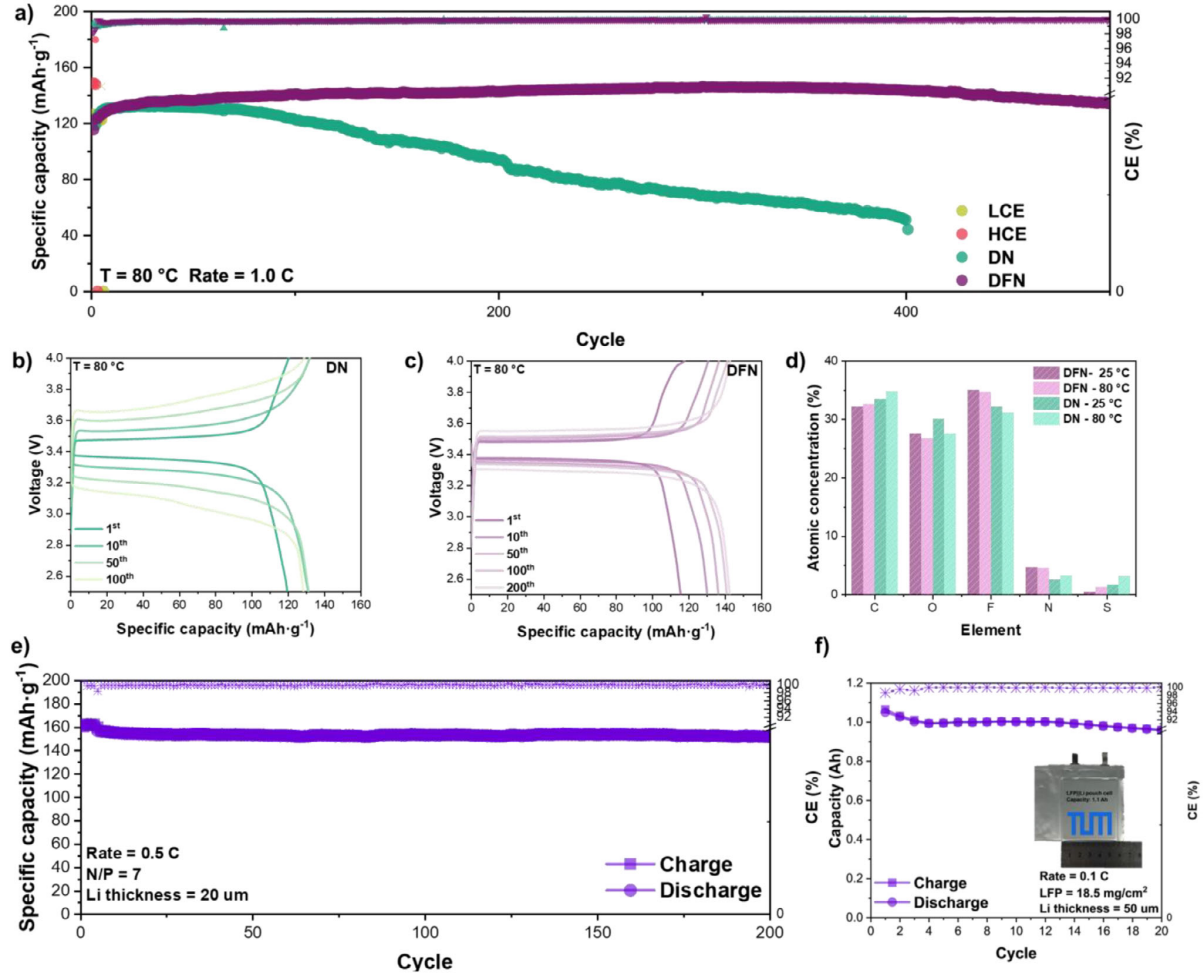
a) Long‐term cycling of LFP||Li half‐cells at a rate of 1.0 C in different electrolytes at 80 °C. b), c) Different cycles’ charge–discharge curves of LFP||Li half‐cells in DN and DFN electrolyte at 80 °C. d) Atomic concentration of CEI at different temperatures with DN and DFN electrolytes. e) Long‐term cycling of LFP||Li (20 µm) full‐cells at a rate of 0.5 C with DFN at room temperature. f) Long‐term cycling of LFP||Li pouch cell at a rate of 0.1 C with DFN at room temperature.

As depicted in Figure 5a, the battery with DFN demonstrates an astonishing performance. After the initial several cycles of formation, the capacity attains 140 mAh g^−1^ and remains stable for more than 500 cycles. At 500 cycles, it still reached 134.4 mAh g^−1^, with a capacity retention rate of 96.0% and an average efficiency as high as 99.81%. By contrast, the performance of LCE and HCE is ineffective at elevated temperatures of 80 °C due to the intensified consumption of FSI^−^ in the electrolyte, leading to vigorous decomposition (Figure S49). Additionally, the DN remains stable initially but exhibits a rapid decay after 70 cycles. By the 400^th^ cycle, the capacity retention rate has significantly declined to 36.7%. The observed phenomenon can be ascribed to the preferential decomposition of lithium nitrate, subsequently leading to an accelerated degradation of the electrolyte. This degradation results in an increase in polarization and a decrease in capacity. Conversely, DFN maintains a stable voltage platform. (Figure 5b,c) To further demonstrate the interfacial stability between the electrolytes and the cathode, we compare the XPS spectra of the cathode after 30 cycles at 25° and 80 °C temperatures. The atomic ratios of O, F, S, and N in the CEI formed at 80 °C exhibit negligible variations when compared to those formed at 25 °C, as depicted in Figure 5d. Irrespective of the temperature conditions, whether elevated or room temperature, DFN exhibits an exceptionally low concentration of S elements. This observation indicates that its solvation structure maintains stability at high temperatures while significantly suppressing the decomposition of anions.

The potential practical application of DFN is further demonstrated by assembling LFP||Li full cells with different N/P ratios–13, 7 and 1.8. As the N/P ratio decreases, the cycling stability of the battery progressively diminishes. At an N/P ratio of 13, DFN can facilitate over 600 cycles without degradation while maintaining a nearly constant voltage platform (Figure S50). Conversely, when the lithium foil thickness is 20 µm (N/P = 7), the battery remains capable of stable cycling for over 200 cycles, achieving a capacity retention rate close to 100% (Figures 5e and S51). Under more extreme conditions, specifically with a reduced lithium thickness of 5 µm and an N/P ratio of only 1.8, the lithium metal full cell exhibits cycling beyond 50 cycles (Figure S52). Importantly, this degradation is not attributed to increased polarization at the voltage platform, highlighting the excellent compatibility between DFN and LMA. Figures 5f and S53 illustrate the long‐term stability of the 1.1 Ah LFP||Li pouch cell with DFN. The battery retains 91.0% of its initial capacity after 20 cycles with 99.82% average CE and maintains a low polarization potential, demonstrating its promising potential for commercialization. Our results confirm that the electrolytes using the weakly solvated structure of glymes demonstrate remarkable electrochemical performance advantages under extreme conditions in the realm of LMBs (Table S4).

## Conclusion

In conclusion, we have successfully developed a novel electrolyte by effectively modulating competitive solvation structures. In this formulation, the C─O─C motifs of glymes are competitively substituted by other anions and solvents to achieve single oxygen site coordination, thereby facilitating a weak solvation effect. At an apparent concentration of 1.25 M, a solvated sheath enriched with anions and single oxygen‐bound complexes is formed, which significantly enhances lithium metal compatibility and promotes rapid desolvation kinetics. The DFN electrolyte exhibits remarkable stability at both 25° and 80 °C, enabling the LFP||Li cell to achieve over 2000 cycles (capacity retention: 90.76%) and 500 cycles (capacity retention: 96%), respectively. Interestingly, the LFP||Li (N/P = 7) full battery affords close to 100% of initial capacity over 200 cycles and exceeds 50 cycles at an N/P ratio of 1.8. Moreover, DFN can enable a 1.1 Ah LFP||Li pouch cell to cycle stably with 91% capacity retention and an average CE of 99.82%, demonstrating significant commercialization potential. The demonstrated distinctive solvation regulation strategy has paved a novel research avenue for the realization of high‐performance LMBs.

## Supporting Information

The authors have cited additional references within the Supporting Information.

## Conflict of Interests

The authors declare no conflict of interest.

## Supporting information



Supporting Information

## Data Availability

The data that support the findings of this study are available from the corresponding author upon reasonable request.
