# Supplementary material for: Anchoring and Competition: Weakly Solvated Structure of Glymes Enhances Stability in Lithium Metal Batteries Operating under Extreme Conditions
Source: Angew Chem Int Ed Engl. 2025 Aug 14;64(40):e202511336. doi: 10.1002/anie.202511336 (PMC12462747; doi:10.1002/anie.202511336)
Supplement: Supplementary file 1 — Supporting Information [file ANIE-64-e202511336-s001.pdf]

Supporting Information

Anchoring and Competition: Weakly Solvated Structure of Glymes Enhances Stability in Lithium Metal Batteries Operating Under Extreme Conditions

Tianle Zheng,[a] † Mengqi Wu,[b] † Jianwei Xiong,[d] † Ming Yang,[a] † Wenzhe Guo,[a] Qihan Zeng,[a] Hongwei Yu,[i] Tonghui Xu,[b,c] Weiping Xie,[b] Yiyao Xiao,[b] Zhuijun Xu, [a] Yuxin Liang, [a] Zerui Li,[a] Ruoxuan Qi,[a] Guangjiu Pan,[a] Xiaotang Shi,[b] Hongbin Zhao,[c] Xiaohong Li,[h] Yongyao Xia,[d,g] Ya-Jun Cheng,*[b,e] Yonggao Xia,*[b,f] Peter Müller-Buschbaum*[a]

[a] T. Zheng, M. Yang, W. Guo, Q. Zeng, Z. Xu, Y. Liang, Z. Li, R. Qi, G. Pan and P. Müller-Buschbaum
Chair for Functional Materials
Department of Physics
TUM School of Natural Sciences
Technical University of Munich
James-Franck-Str. 1, 85748 Garching, Germany
E-Mail: muellerb@ph.tum.de

[b] M. Wu, T. Xu, W. Xie, Y. Xiao, X. Shi, Y.-J. Cheng and Y. Xia
Ningbo Institute of Materials Technology and Engineering
Chinese Academy of Sciences
1219 Zhongguan West Rd, Ningbo, 315201, Zhejiang Province, P. R. China
E-Mail: chengyj@nimte.ac.cn; xiayg@nimte.ac.cn

[c] T. Xu, H. Zhao
Department of Chemistry
College of Sciences
Shanghai University
Shanghai, 200444, P. R. China

[d] J. Xiong, Y. Xia
College of Materials Science and Technology
Nanjing University of Aeronautics and Astronautics
Nanjing, 210016, P. R. China

[e] Y.-J. Cheng
College of Renewable Energy
Hohai University
1915 Hohai Ave, Jintan District, Changzhou, Jiangsu Province, 213220, P. R. China
E-Mail: chengyj@nimte.ac.cn

[f] Y. Xia
Center of Materials Science and Optoelectronics Engineering
University of Chinese Academy of Sciences
19A Yuquan Rd, Shijingshan District, Beijing 100049, P. R. China
E-Mail: xiayg@nimte.ac.cn

[g] Y. Xia
Department of Chemistry
Institute of New Energy
Fudan University
Shanghai, 200433, P. R. China

[h] X. Li
College of Chemistry

Chemical Engineering and Materials Science
Soochow University
Suzhou, Jiangsu, China, 215123

[i] H. Yu
School of Materials Science and Chemical Engineering
Ningbo University
Ningbo 315211, P. R. China.

† T. Zheng, M. Wu, J. Xiong and M. Yang contributed equally to this work.

Methods

Materials

Lithium nitrate ($LiNO_3$, 99.99% trace metals basis), lithium hexafluorophosphate ($LiPF_6$, 99.95%), poly (vinylidene fluoride) (PVDF), diethylene glycol dimethyl ether (G2, anhydrous, 99.5%), and N-methyl-2-pyrrolidone (NMP, anhydrous, 99.5%) were procured from Sigma-Aldrich. Conductive carbon black (Super P) and lithium iron phosphate (LFP) powder were obtained from NEWARE TECHNOLOGY LIMITED. Fluoroethylene carbonate (FEC, 99.9%) and lithium bis(fluorosulfonyl)imide (LiFSI, 99.95%) were supplied by Guotai-Huarong Commercial New Material Co., Ltd. Lithium foil with a thickness of approximately 200 μm was sourced from Dongguan Shanshan Battery Materials Co., Ltd. All chemicals were used as received without further purification.

Electrolyte Solution Preparation and Electrode Fabrication

The present study prepared five distinct electrolyte solutions: 1.0 M LiFSI in G2 solvent (LCE); 2.0 M LiFSI in G2 solvent (HCE, the maximum achievable concentration is approximately 2.0 M); 1.0 M LiFSI and 0.5 M $LiNO_3$ in G2 solvent (DN); 1.0 M LiFSI and 0.25 M $LiNO_3$ in a G2/FEC cosolvent (v/v = 1:1) (DFN). (In DFN, FEC functions as a pseudo-diluent for $LiNO_3$, effectively reducing the concentration of $LiNO_3$ dissolved in G2/FEC to approximately 0.25 M, which is equivalent to a solution of 0.5 M $LiNO_3$ in G2.)

All electrolytes were thoroughly mixed under continuous stirring at 60 °C for 4 hours at a rotational speed of 1000 rpm inside an Ar-filled glove box. Prior to electrode fabrication, LFP powders were vacuum-dried at 80 °C for 8 hours. The dried powders were subsequently mixed with Super P and PVDF in a mass ratio of 8:1:1 in NMP. Continuous stirring was performed for 12 hours to ensure complete dissolution of PVDF and homogeneous mixing of the powders. The resulting slurries were cast onto Al or Cu current collectors, achieving mass loading densities of approximately 5.0 to 7.0 $mg \cdot cm^{-2}$ of active material. Li||Li, Li||Cu, and Li||LFP cells were assembled in CR 2032-type coin cells using a specific electrolyte solution volume of 60 μl. Cell assembly and electrolyte injection procedures were conducted within an O_2 and H_2O level-controlled glove box (MBRAUN UniLab) with concentrations below 0.1 ppm.

Electrochemical Tests

The Li||Cu cells employed a 14 mm diameter Cu foil as the working electrode, while Li metal foils were utilized as the reference and counter electrodes. In each cycle, a predetermined capacity of Li was deposited onto the Cu foil at a specific current density and subsequently stripped until the potential

reached 0.5 V (vs. Li^+/Li). Symmetric Li||Li cells were used to investigate cycling stability under various current densities and electrolytes at a specific temperature.

Long-term cycling and rate tests were performed on the Li||LFP battery with a voltage range of 2.5 V to 4.0 V (vs. Li^+/Li). To investigate the electrolyte evolution and reactions at extremely high temperatures on both sides of the cathode and anode, Li||LFP half cells with various electrolytes were cycled at 1.0 C (1.0 C = 170 $mAh \cdot g^{-1}$) in an oven.

Cyclic voltammetry measurements (2.5 V - 4.0 V of LFP, 0 V - 2.5 V of Cu, and -0.1 V to 0.1 V of Li symmetric anode) and electrochemical impedance spectroscopy tests (0.01 Hz - 1 MHz, 10 mV) were performed using an electrochemical workstation (Solartron Analytical). At the discharge end, all the cells were tested at the charge-discharge or discharge-charge cycle end.

In accordance with a previously reported methodology, the Li/Li symmetric cell architecture was used to estimate the migration number of lithium ions (t_{Li+}) in the electrolyte.[1] EIS experiments were conducted both before and after the current reached a steady state by applying a minute polarization potential of 10 mV and frequency range from 1 MHz – 0.01 Hz to the symmetric cell (with a relaxation time of 3000s). The following equation was utilized for calculating t_{Li+}.

$$t_{Li^+} = \frac{I_s(\Delta V - I_o R_o)}{I_o(\Delta V - I_s R_s)}$$

where I_o and I_s represent the current of the initial and stable state after polarization, respectively. R_o and R_s express the interfacial resistance values of the lithium electrode before and after polarization. ΔV is the potential of polarization.

Characterization of Electrodes and Electrolyte Solution

The Fourier transform infrared spectroscopy (FTIR, NICOLET 6700) analysis was conducted at a temperature of 25 °C. Raman spectra of the electrolytes were acquired using a Renishaw in Via Reflex Raman microscopy system at ambient temperature. Scanning electron microscopy (SEM) images were obtained from a Hitachi S4800 cold-field emission scanning electron microscope equipped with energy-dispersive X-ray spectroscopy (EDS). For XPS and TOF-SIMS measurements, Cu electrodes retrieved from Li ||Cu cells after one cycle of plating/stripping at 0.5 mA cm^{-2} and 3 mAh cm^{-2} were transferred to instrumental chambers from an Ar-filled glove box without exposure to air. XPS depth profiles were collected on a Nexsa instrument (Thermo Fisher Scientific) using a monochromatic Al Ka X-ray source (excitation energy = 1468.6 eV) and Ar^+ sputtering for different durations: 0, 10, 30, and 60 s. TOF-SIMS analyses were performed on a TOF-SIMS 5-100 instrument (IONTOF GmbH) in high mass resolution

mode utilizing a Bi_3^+ ion beam with an energy of 30 keV for the acquisition phase and Cs^+ beam with an energy of 1 keV for the sputter phase. The typical areas analyzed by TOF-SIMS were measured as follows: the acquisition phase covered an area of 50 μm ×50 μm while the sputter phase covered an area of 200 μm ×200 μm.

Evolution of in-situ ATR-FTIR experiment

In-situ ATR-FTIR experiments were acquired with a Thermo Fisher-Nicolet 6700 FTIR spectrometer equipped with an electrochemical cell having two electrodes. The electrochemical cell used a diamond crystal as the IR window. The cell is made of polyether ether ketone material (PEEK). All spectra were acquired with a resolution of 4 cm^{-1}. The spectra are presented in the form of absorbance $\boldsymbol{A} = \boldsymbol{log}(\boldsymbol{1}/\boldsymbol{T}) = -\boldsymbol{log}(\boldsymbol{R_s}/\boldsymbol{R_{ref}})$, where R_s and R_{ref} are the single-beam spectrum of the sample charged state and open-voltage state, respectively.

Calculation Method

Density functional theory (DFT) calculations were performed using the Dmol3 module in Material Studio software with the B3LYP functional, custom Grimme DFT-D2 parameters, and DNP 4.4 basis set. [2] ESP charges were applied to optimize all molecules. [3]The k-points were set at Gamma (1 × 1 × 1), and the convergence tolerance was set at 1.0 × 10^{-6} Ha, 2.0 × 10-3 Ha/Å-1, and 5.0 × 10-3 Å for energy, maximum force, and maximum displacement, respectively.

Molecular dynamics (MD) simulations involved the construction of four electrolyte models: a low concentration electrolyte (LCE) comprising 40 LiFSI and 280 G2 molecules; a high concentration electrolyte (HCE) comprising 80 LiFSI and 280 G2 molecules; the DN electrolyte consisting of 40 LiFSI, 20 $LiNO_3$, and 280 G2 molecules; and the DFN electrolyte containing 40 LiFSI, 10 $LiNO_3$, 280 FEC, and 140 G2 molecules. All simulations were performed using the Forcite module with the COMPASS force field. However, it should be noted that in accordance with previous studies, the charges of cations and anions were scaled by a factor of 0.7. [4] The equilibration process for solution models was carried out in the NPT ensemble using a Berendsen barostat with a decay time of 0.1 ps for a duration of 20 ps at a temperature maintained by the Nose thermostat set to a constant value of 298 K with 0.1 GPa pressure. [5] Subsequently, production runs were conducted in an NVT ensemble over a period of 200 ps to ensure equilibrium within the solution system.

The DMol3 code was also used to perform *Ab-initio* molecular dynamic (AIMD) simulations, using the GGA/PBE functional, in order to elucidate the atomic or molecular mechanism. [6] A typical solvation

structure of DFN on the lithium metal surface was introduced into the simulation box. The AIMD simulations were conducted in the NVT ensemble with a Nose-Hoover thermostat and a time step of 1 fs at 298 K for a duration of 10 ps. All calculations utilized the DNP 4.4 basis set for reliable results, with an orbital cut-off set at 4.4. To enhance computational efficiency, an energy convergence criterion of 1.0 × 10^{-5} HA was applied for SCF density convergence.

Supporting Figures

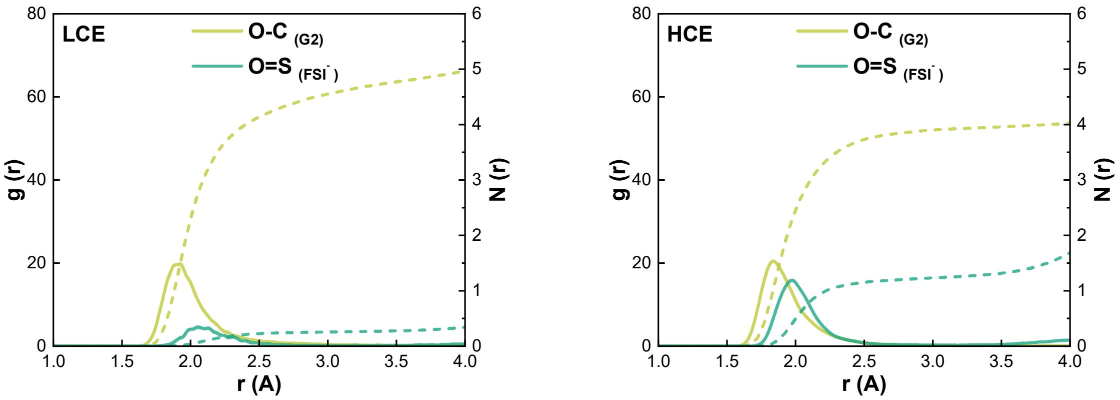

Figure S1. RDF results of LCE and HCE.

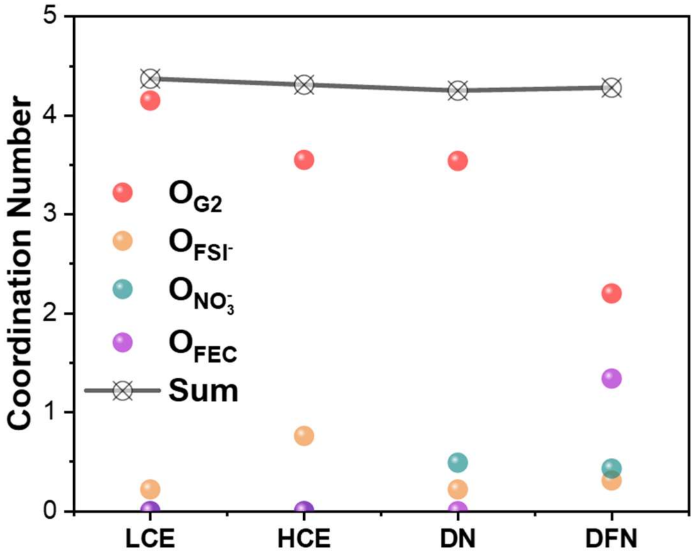

Figure S2. Coordination number of different atoms in various electrolytes.

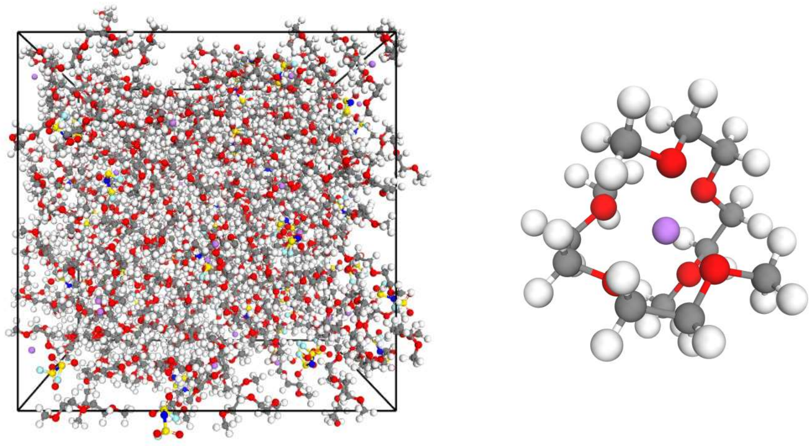

Figure S3. (left) Snapshot of the electrolyte's structure of LCE; (right) typical solvation structure in LCE.

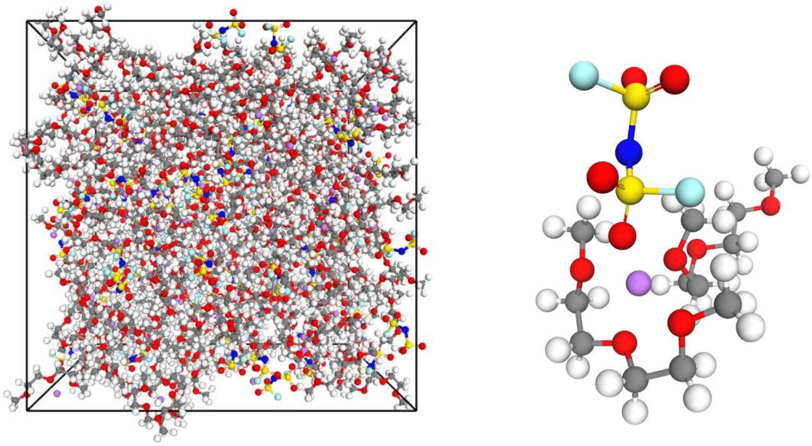

Figure S4. (left) Snapshot of the electrolyte's structure of HCE; (right) typical solvation structure in HCE.

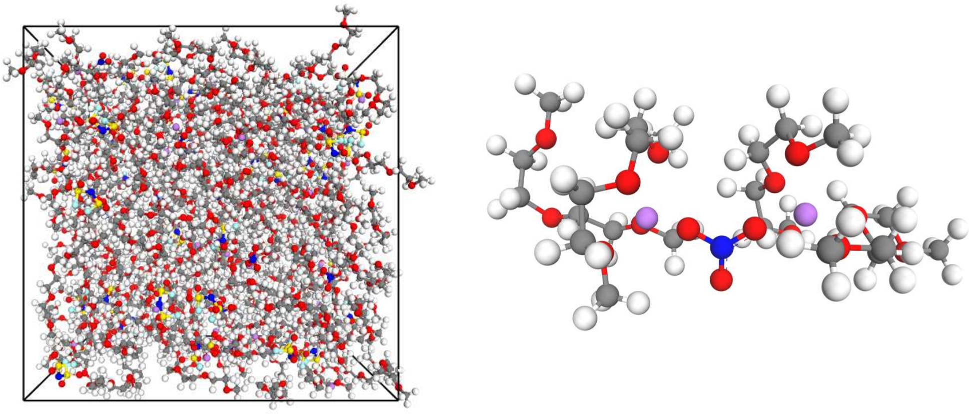

Figure S5. (left) Snapshot of the electrolyte's structure of DN; (right) typical solvation structure in DN.

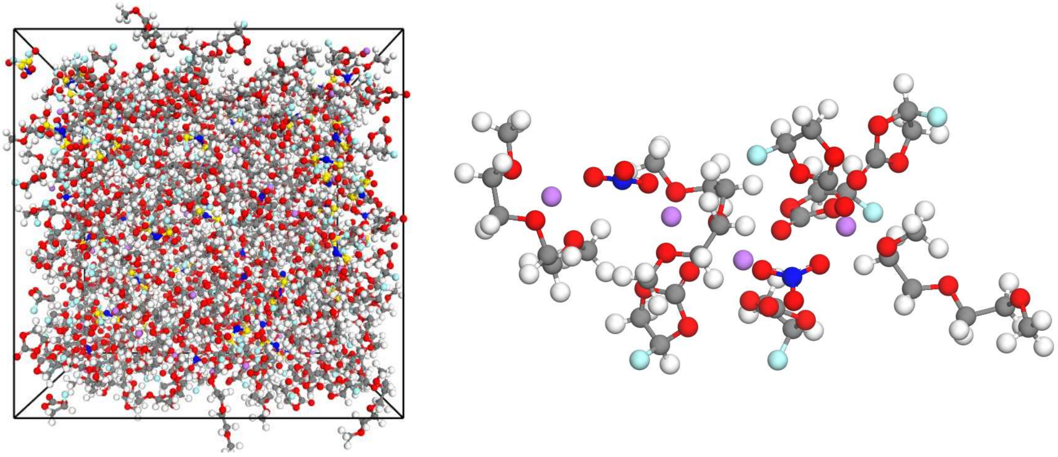

Figure S6. (left) Snapshot of the electrolyte's structure of DFN; (right) typical solvation structure in DFN.

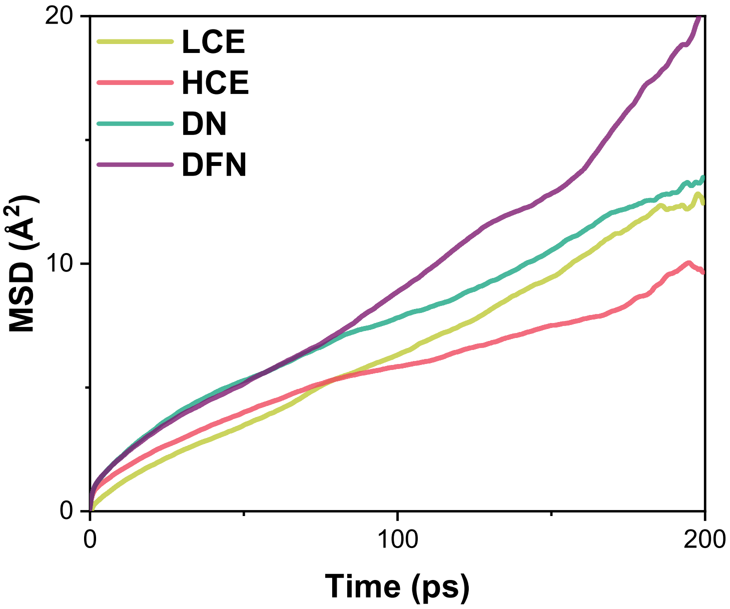

Figure S7. MSD results from different electrolytes.

When multiple oxygen atoms in G2 coordinate with Li^+, the resulting strong binding energy can impede electrolyte kinetics. In DFN, NO_3^- anchors Li^+ on one side while FEC competitively coordinates with Li^+ on the other side. Consequently, G2 coordinates with Li^+ through only a single oxygen site, which reduces the overall desolvation energy barrier. Furthermore, this structure, containing a single O–Li^+ group, allows the remaining oxygen atoms to coordinate with additional Li^+, thereby increasing the number of CIP/AGG structures in the electrolyte and enhancing both the overall Li^+ transference number and diffusion coefficients.

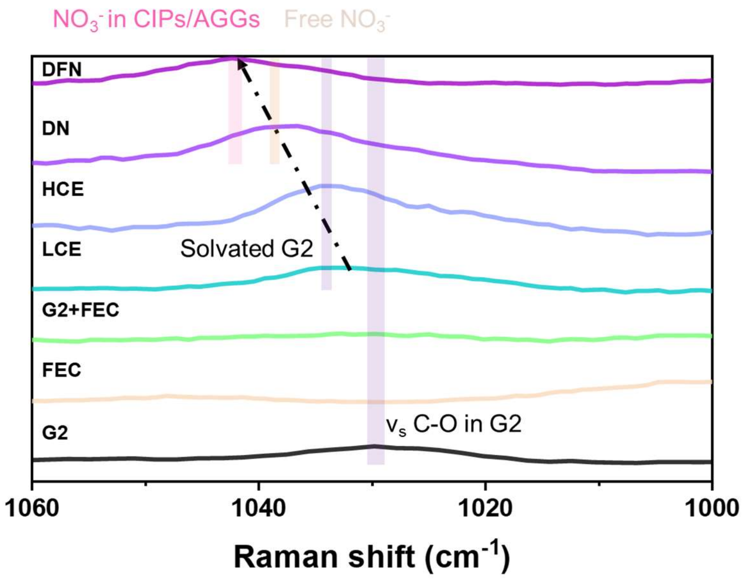

Figure S8. Raman spectra of different electrolytes and solvents in 1000 – 1060 cm^{-1}.

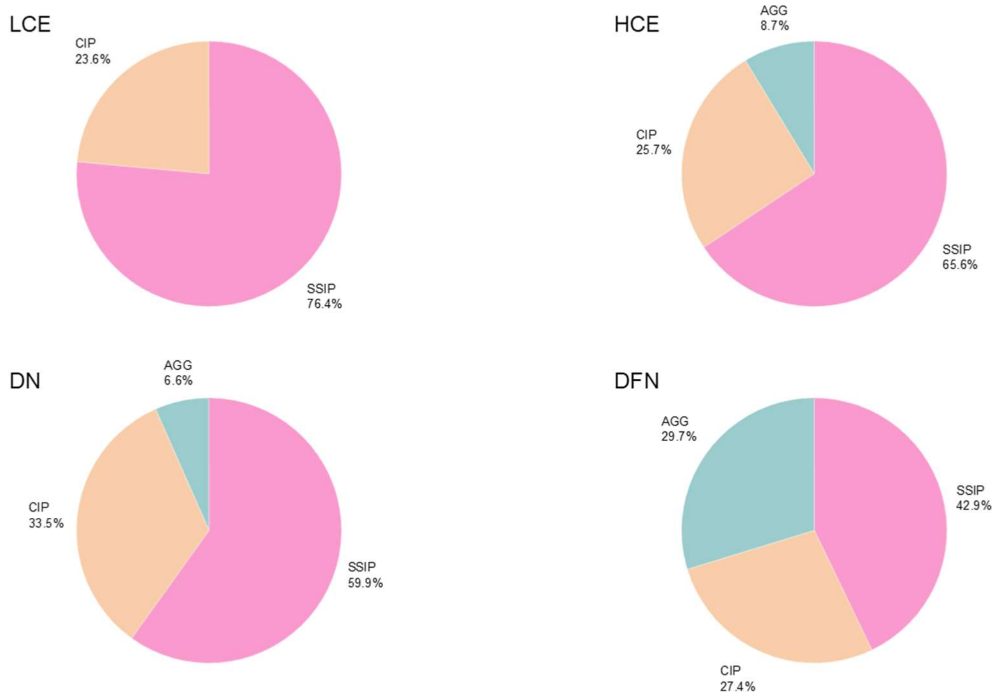

Figure S9. Area ratio of SSIP, CIP, and AGG in different electrolytes.

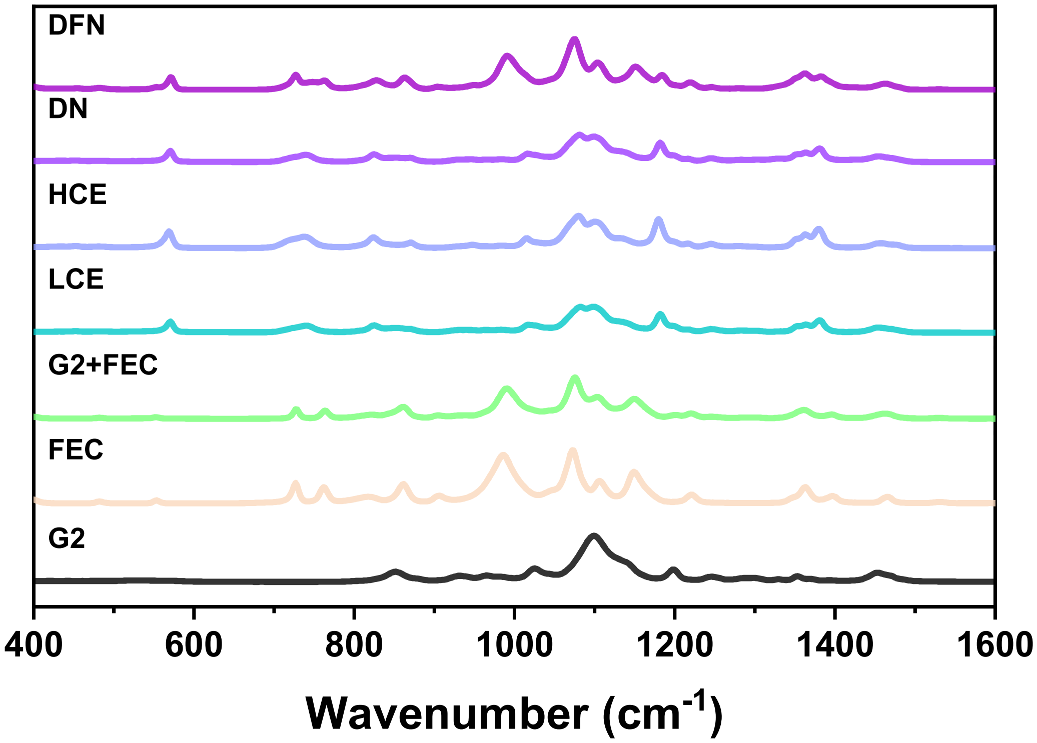

Figure S10. FTIR spectra of different electrolytes and solvents.

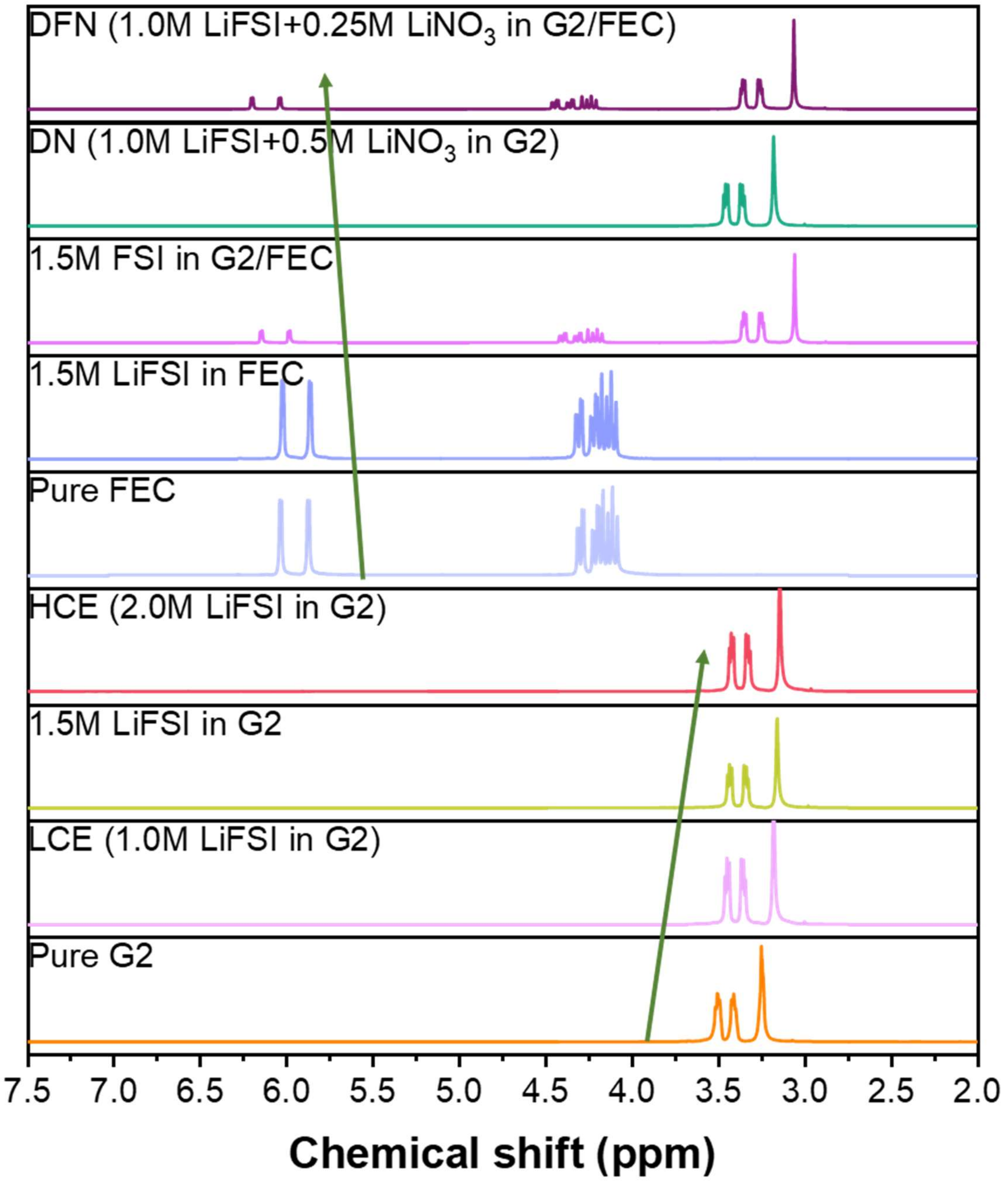

Figure S11. ^{1}H NMR of various electrolytes and solvents.

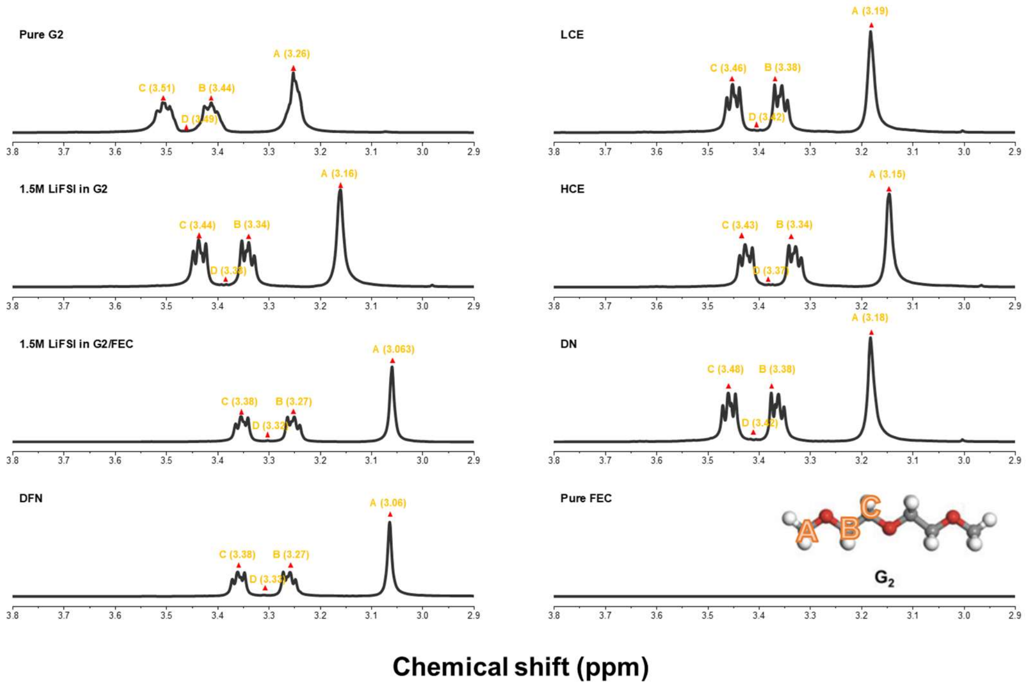

Figure S12. ^{1}H NMR details of various electrolytes and solvents.

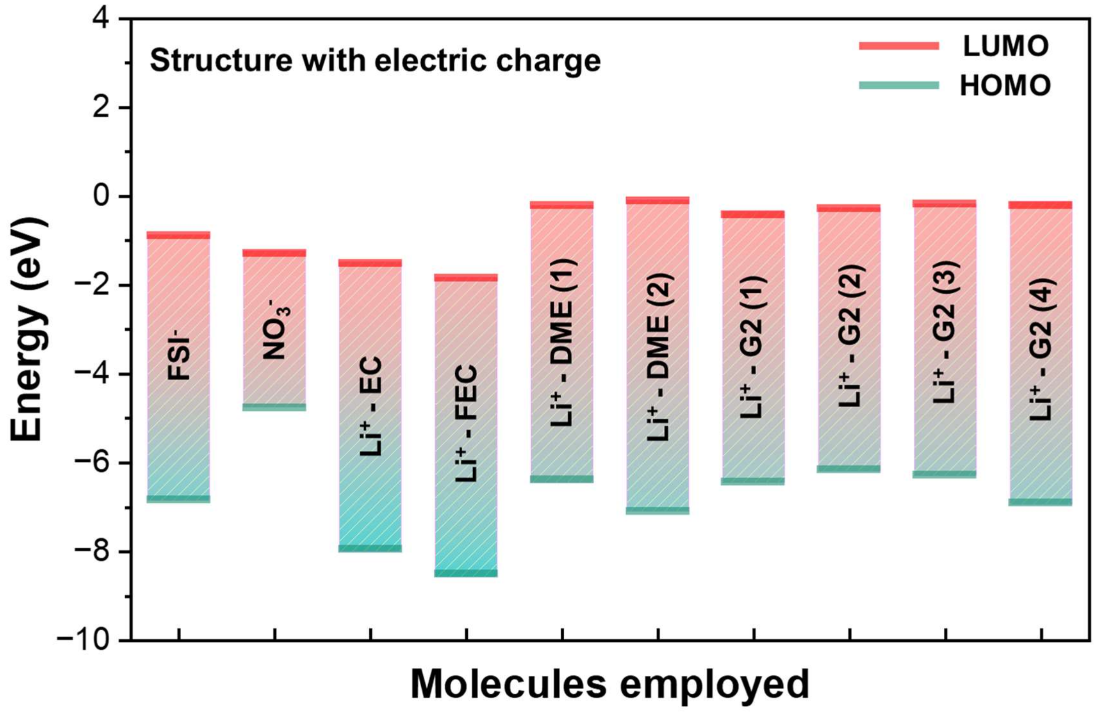

Figure S13. LUMO/HOMO energy level of different structures with charge. (Specific structures in Table S1)

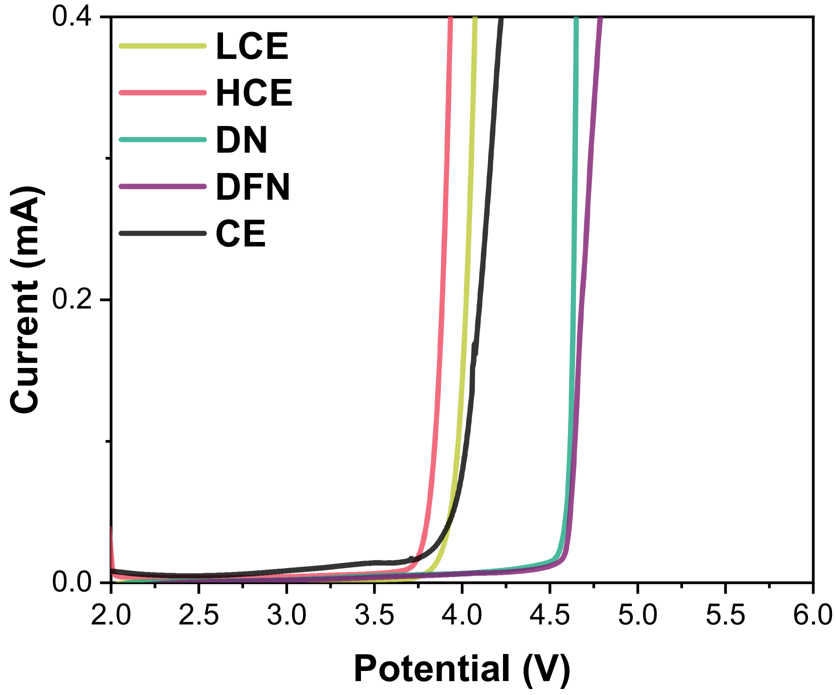

Figure S14. LSV of different electrolytes.

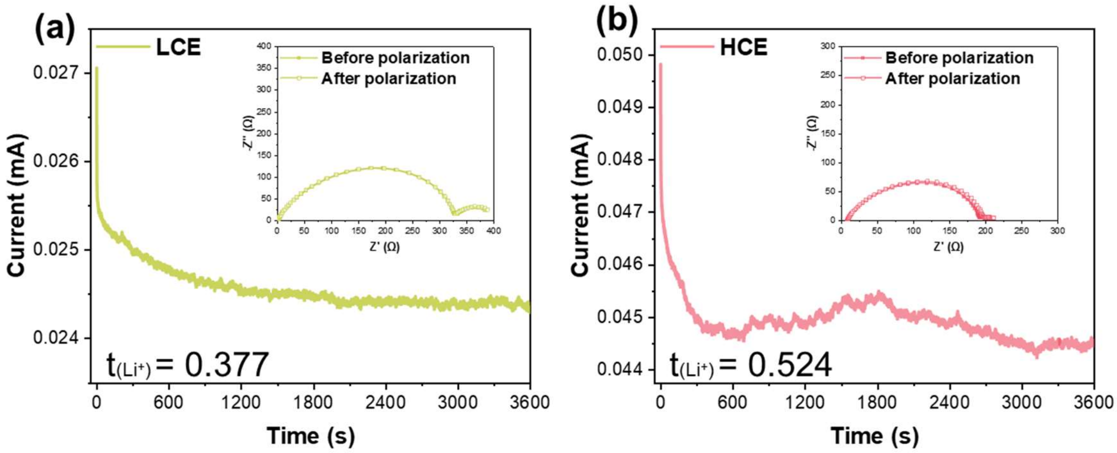

Figure S15. Li^+ transference number of LCE and HCE.

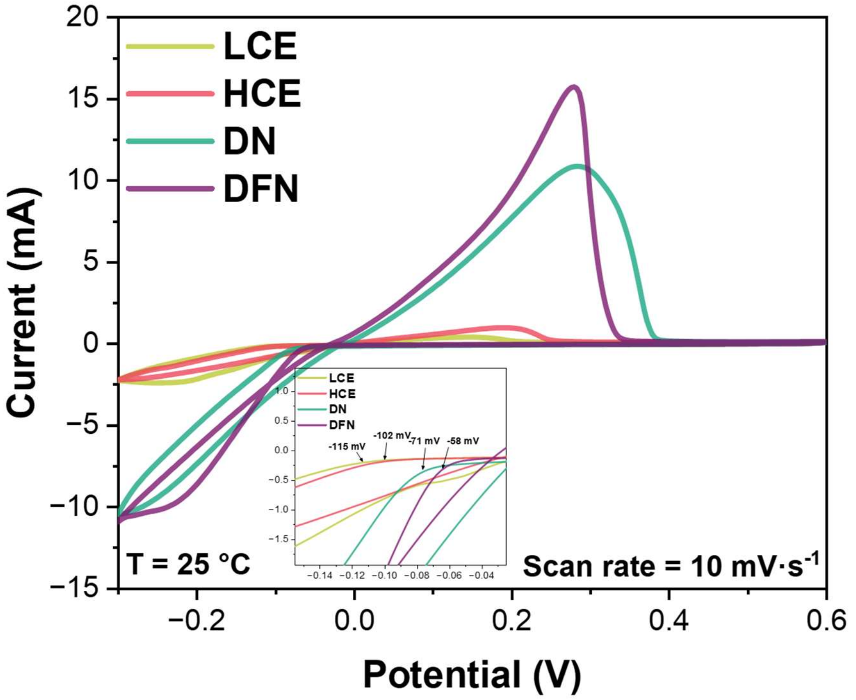

Figure S16. CV curves of -0.3 – 0.6 V at a scan rate of 5 mV s^{-1}

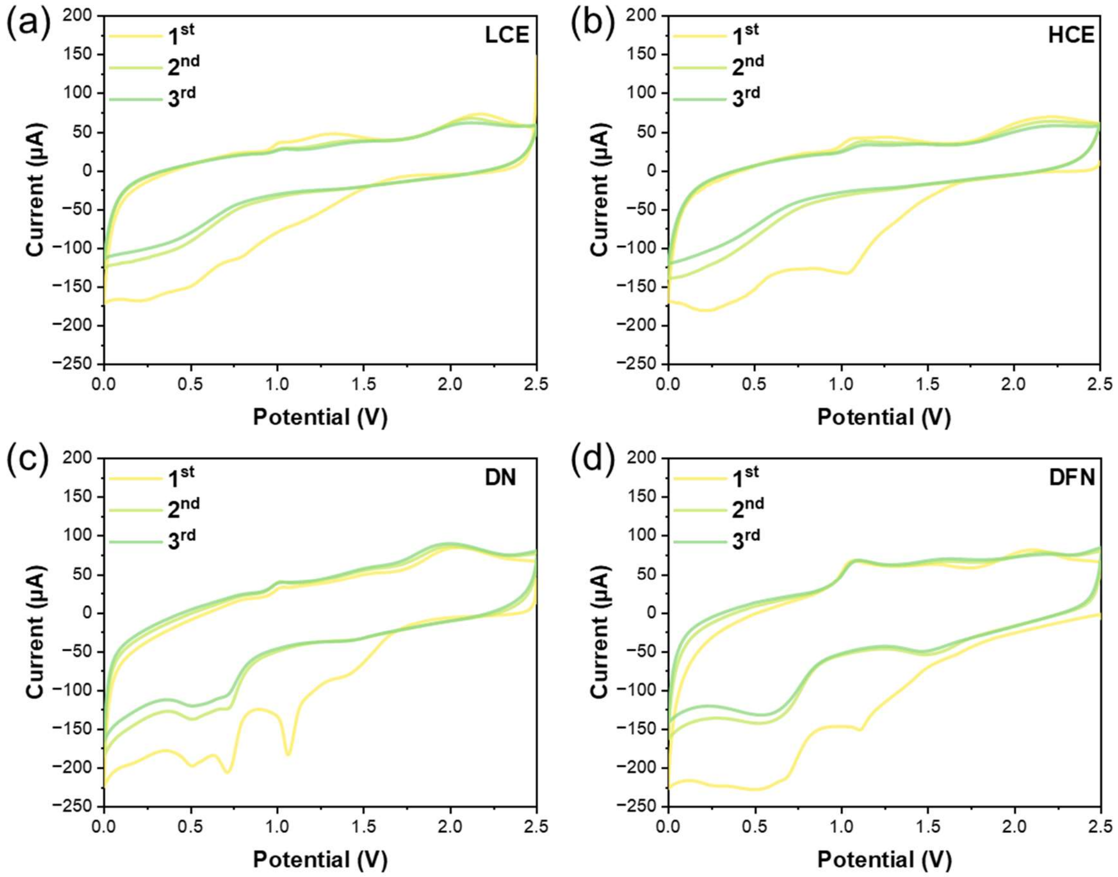

Figure S17. CV curves of 0 – 2.5 V at a scan rate of 5 mV s^{-1} in different electrolytes.

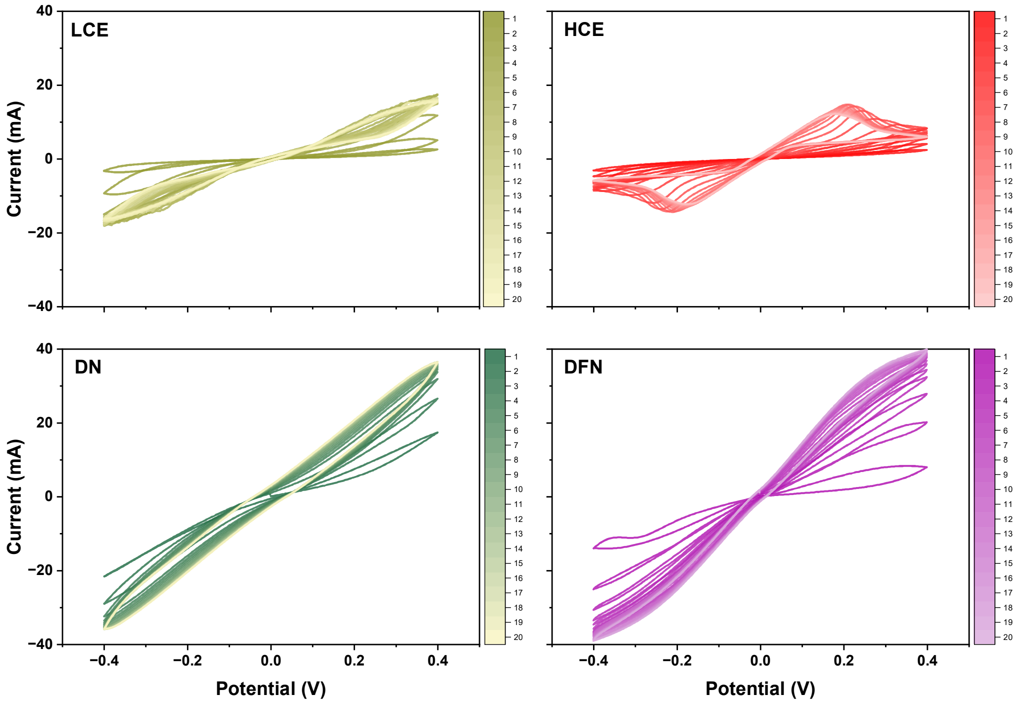

Figure S18. CV curves of -0.4 – 0.4 V of Li||Li symmetric cells at a scan rate of 5 mV s^{-1} in different electrolytes.

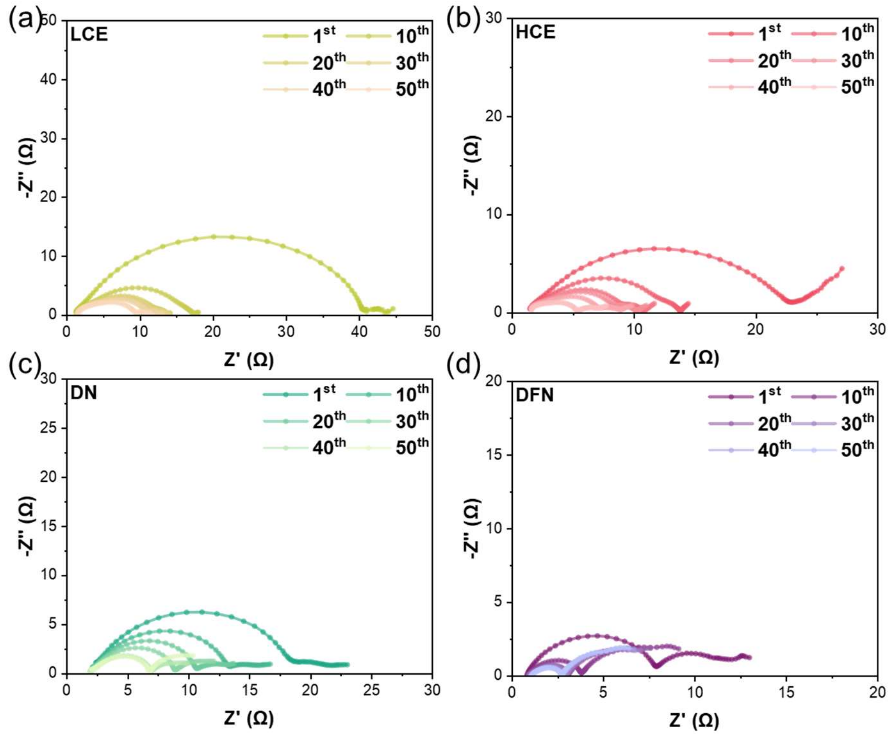

Figure S19. Nyquist plots of electrochemical impedance spectra for Li||Li symmetric cells after different cycles.

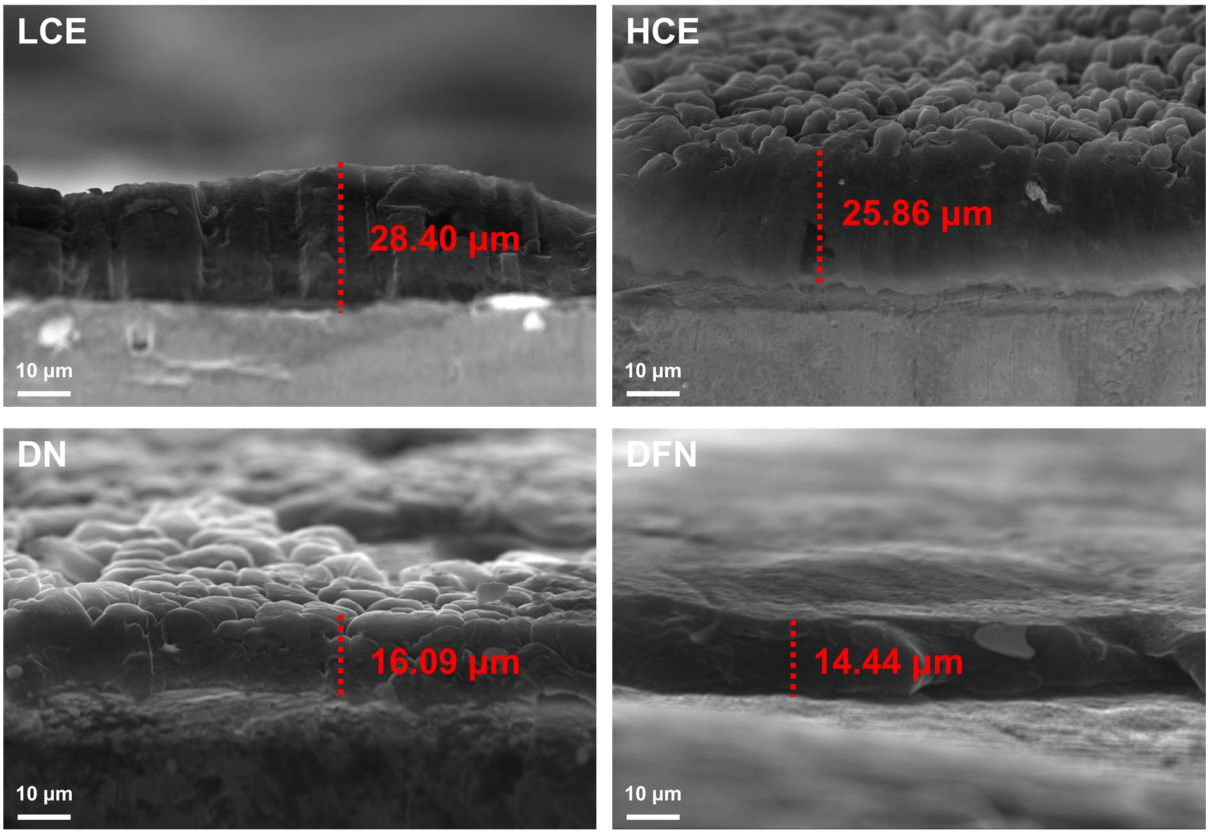

Figure S20. SEM images for the cross-sectional profile of Li electrodeposits after the first plating on the Cu foil at 0.5 mA cm^{-2} and 3 mAh cm^{-2} in different electrolytes.

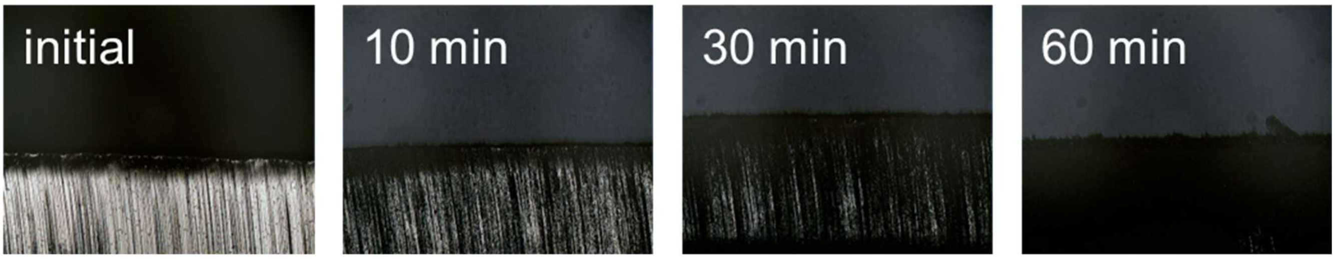

Figure S21. In situ electrochemical optical-microscope images of LCE

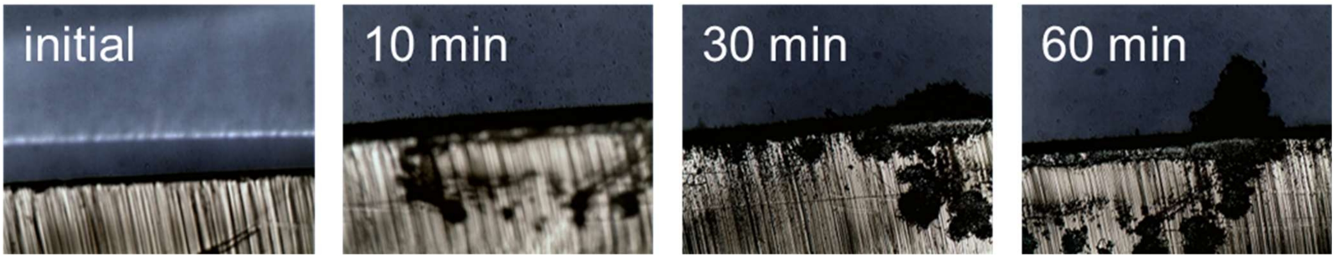

Figure S22. In situ electrochemical optical-microscope images of HCE

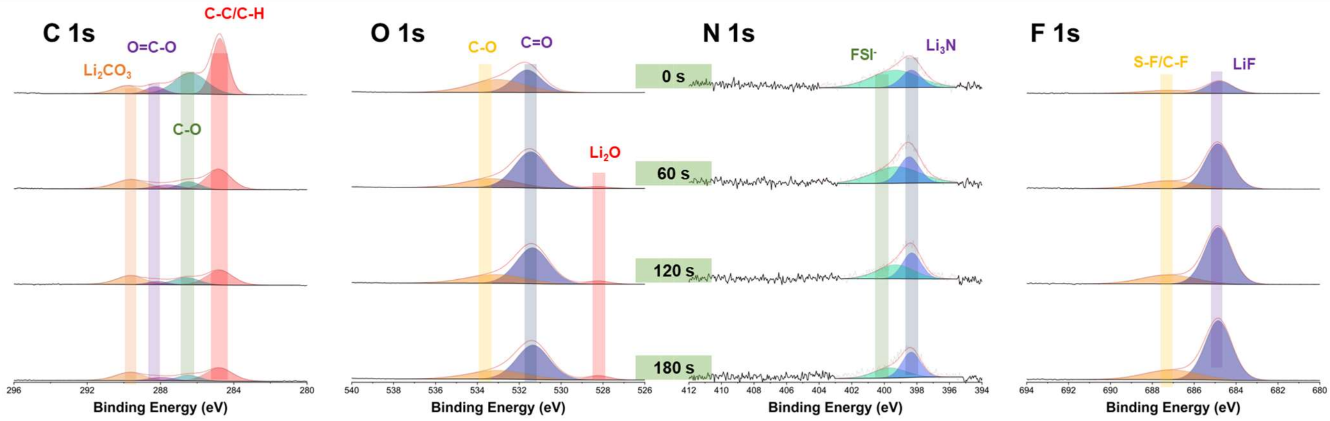

Figure S23. XPS of Li metal anode with LCE

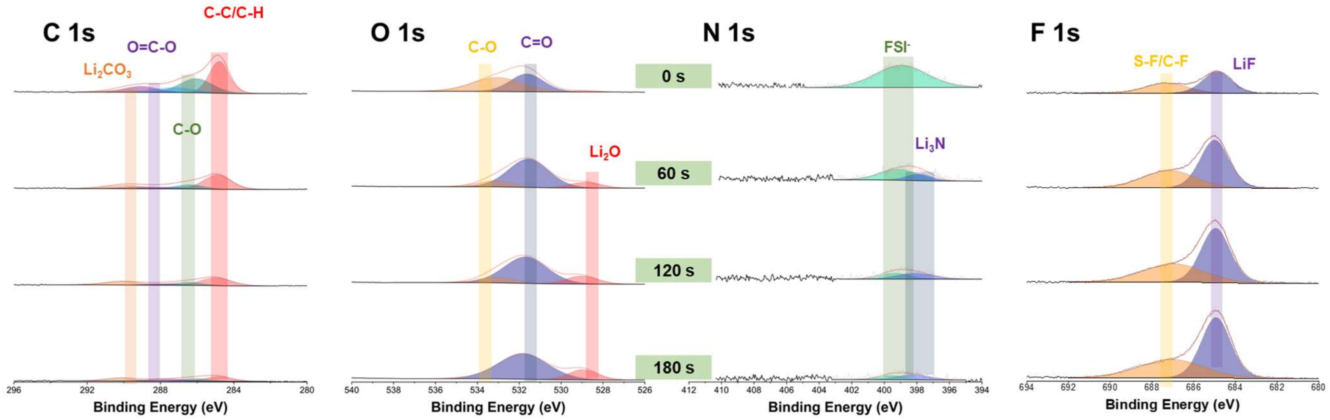

Figure S24. XPS of Li metal anode with HCE

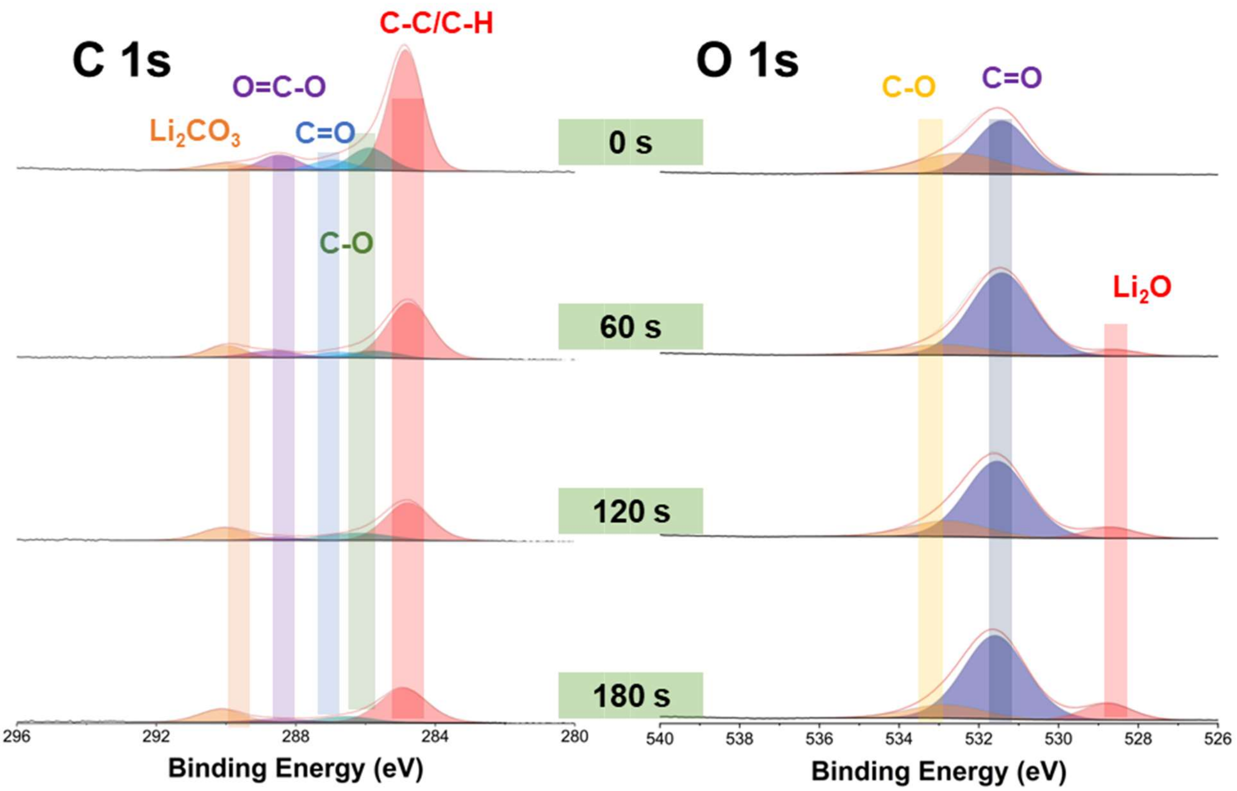

Figure S25. XPS of Li metal anode with DN

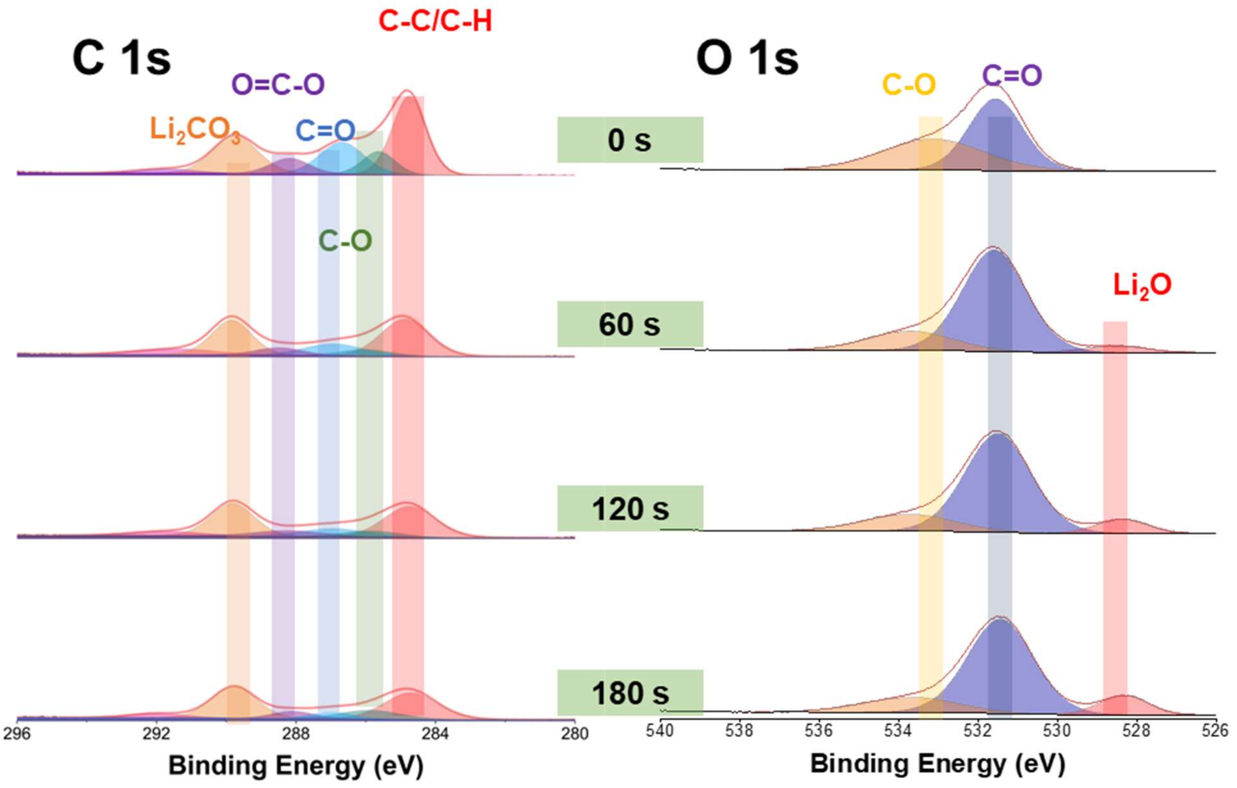

Figure S26. XPS of Li metal anode with DFN

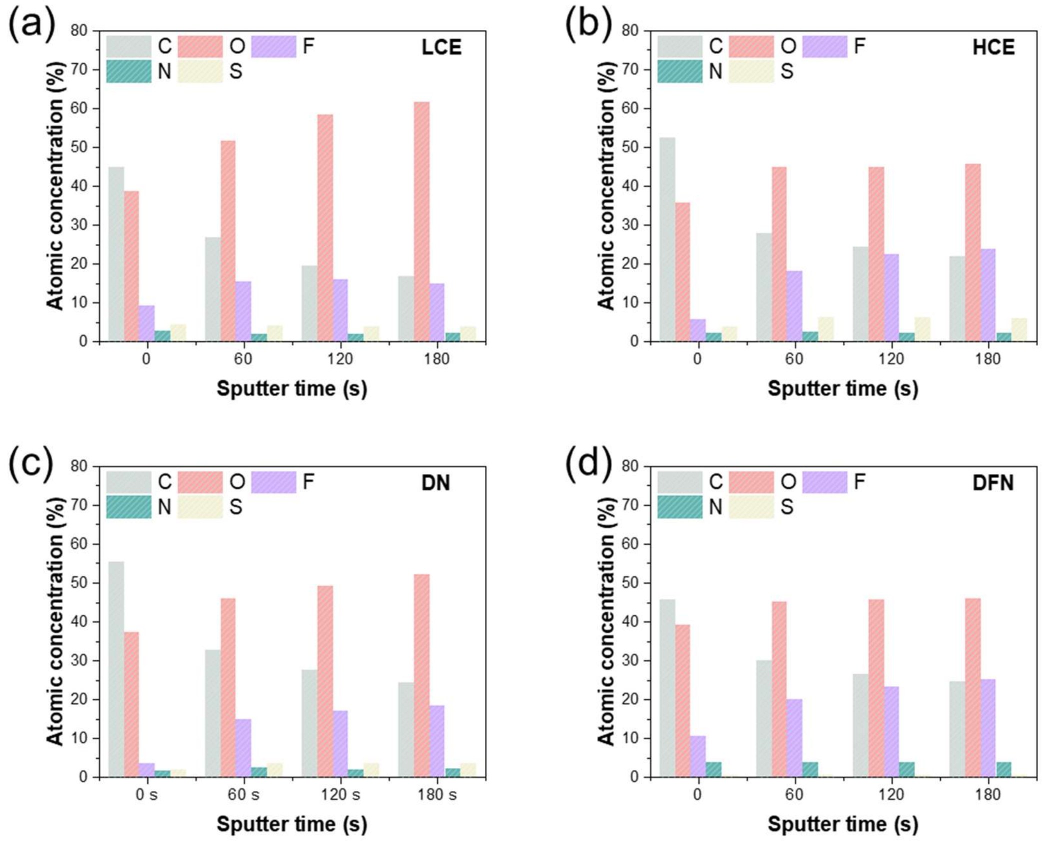

Figure S27. Atomic concentration of SEI in different electrolytes.

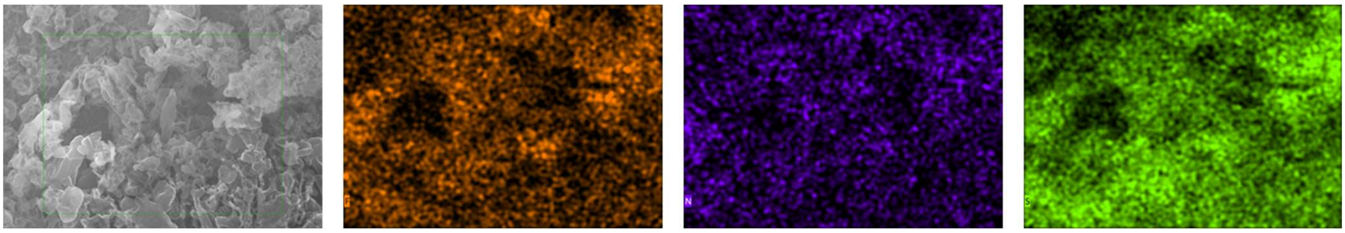

Figure S28. EDS of Li metal anode with LCE (Element: F - Orange, N - Blue, S - Green)

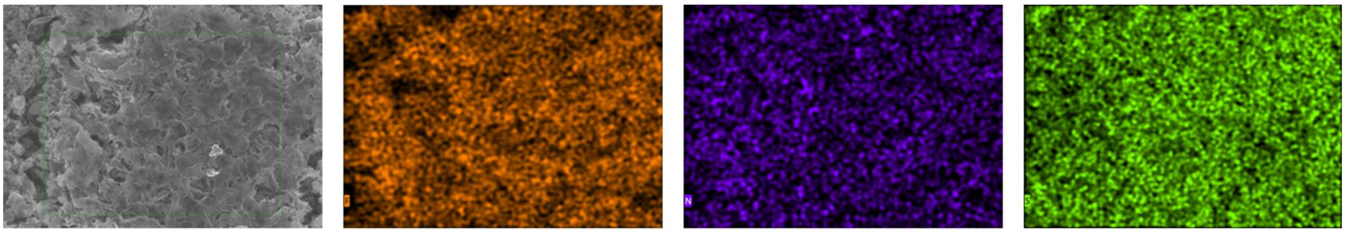

Figure S29. EDS of Li metal anode with HCE (Element: F - Orange, N - Blue, S - Green)

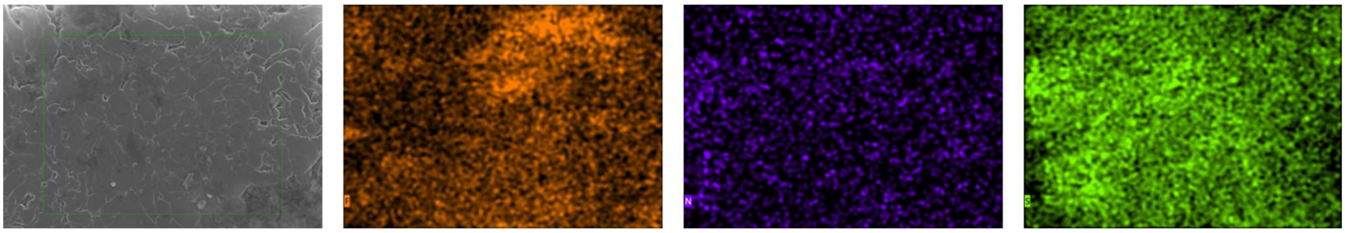

Figure S30. EDS of Li metal anode with DN (Element: F - Orange, N - Blue, S - Green)

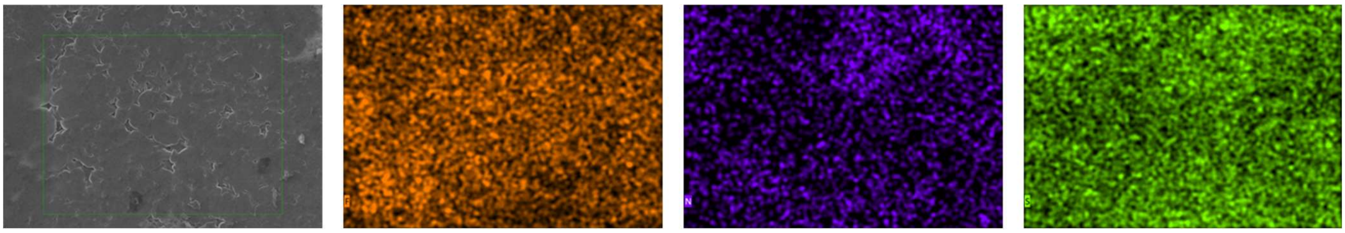

Figure S31. EDS of Li metal anode with DFN (Element: F - Orange, N - Blue, S - Green)

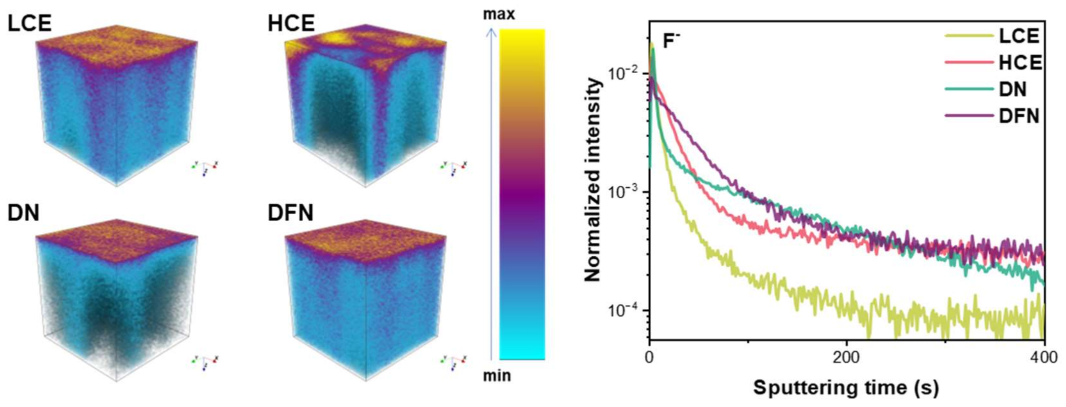

Figure S32. 3D reconstruction for the sputtered volume and composition distribution of 30-cycled Li metal anode for F^-.

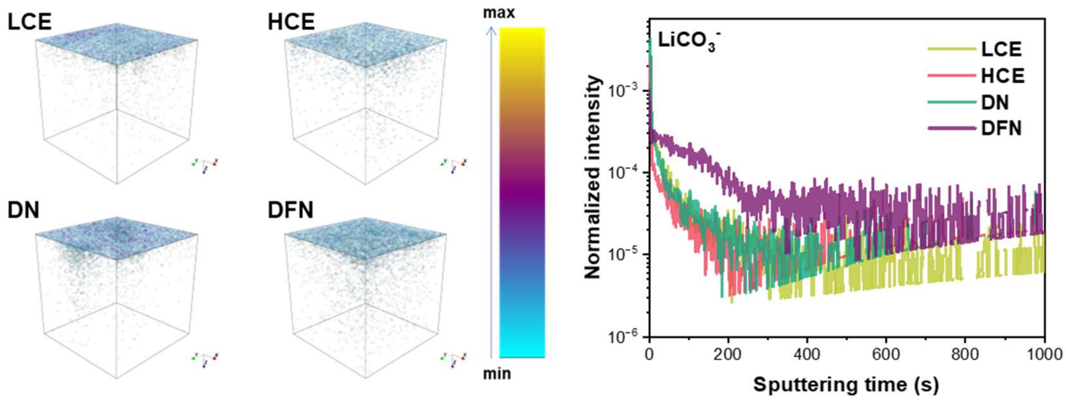

Figure S33. 3D reconstruction for the sputtered volume and composition distribution of 30-cycled Li metal anode for $LiCO_3^-$.

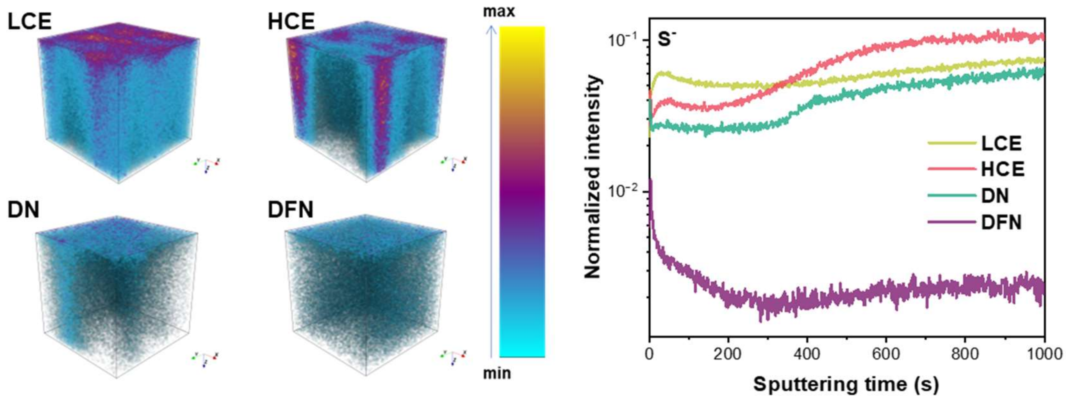

Figure S34. 3D reconstruction for the sputtered volume and composition distribution of 30-cycled Li metal anode for S^-.

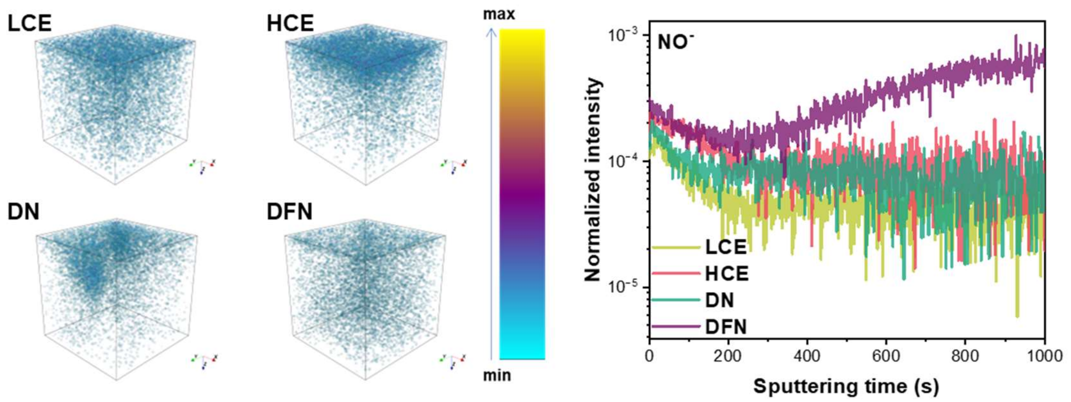

Figure S35. 3D reconstruction for the sputtered volume and composition distribution of 30-cycled Li metal anode for NO^-.

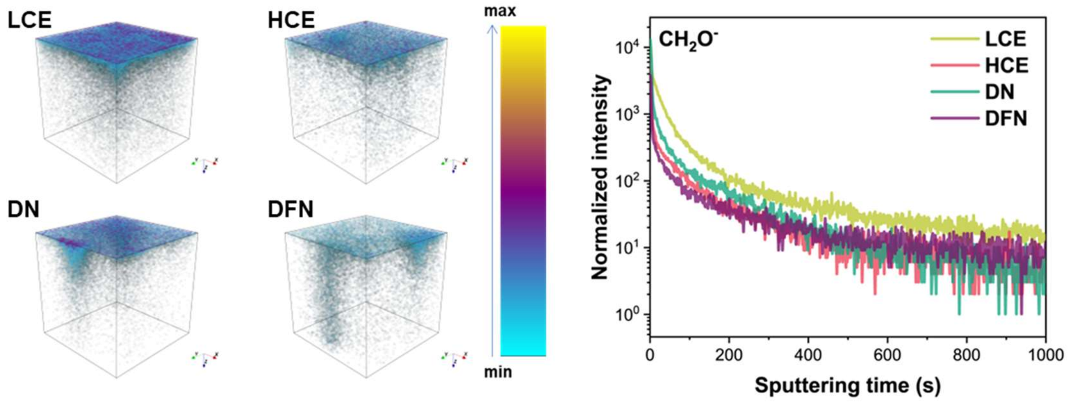

Figure S36. 3D reconstruction for the sputtered volume and composition distribution of 30-cycled Li metal anode for CH_2O^-.

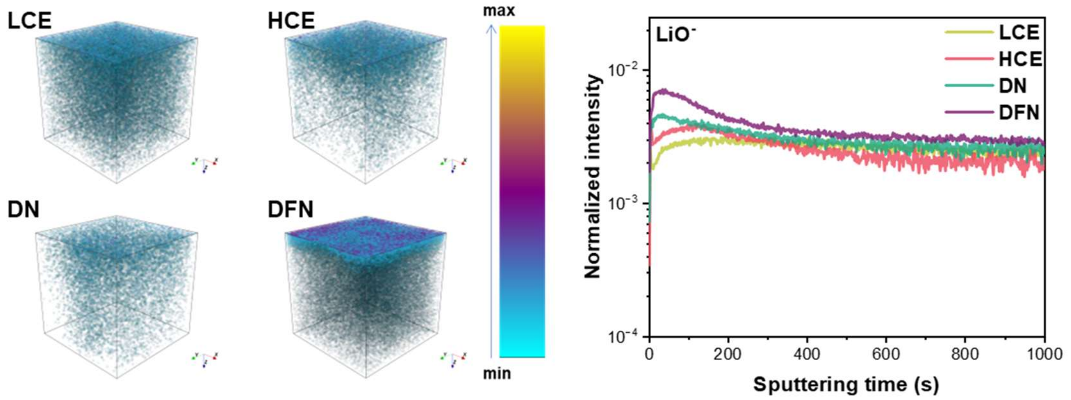

Figure S37. 3D reconstruction for the sputtered volume and composition distribution of 30-cycled Li metal anode for LiO^-.

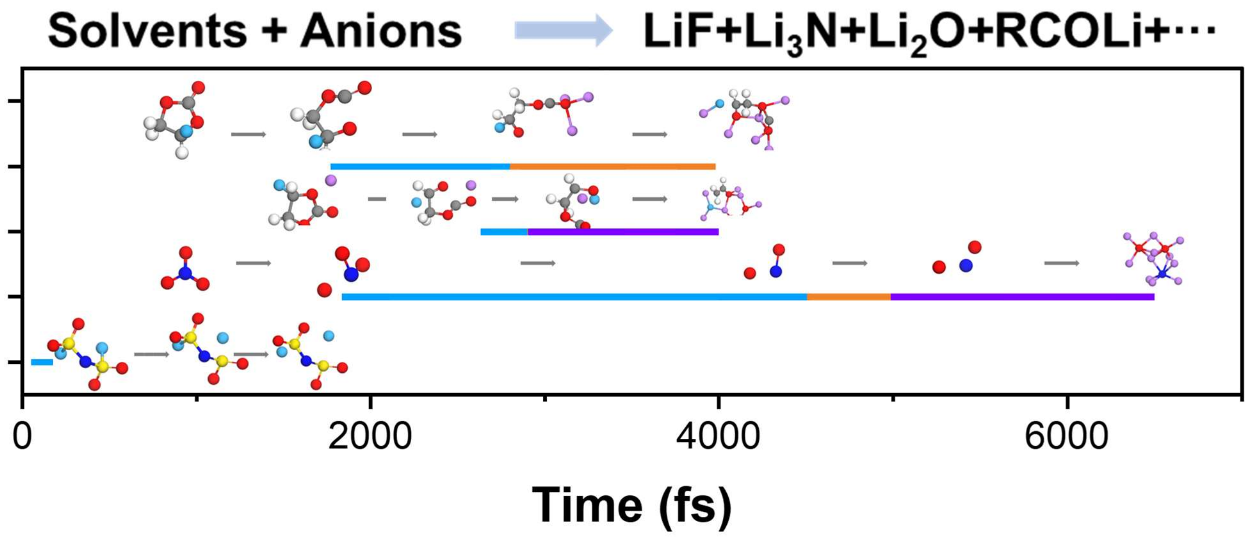

Figure S38. Evolution of different molecule reactions results from AIMD.

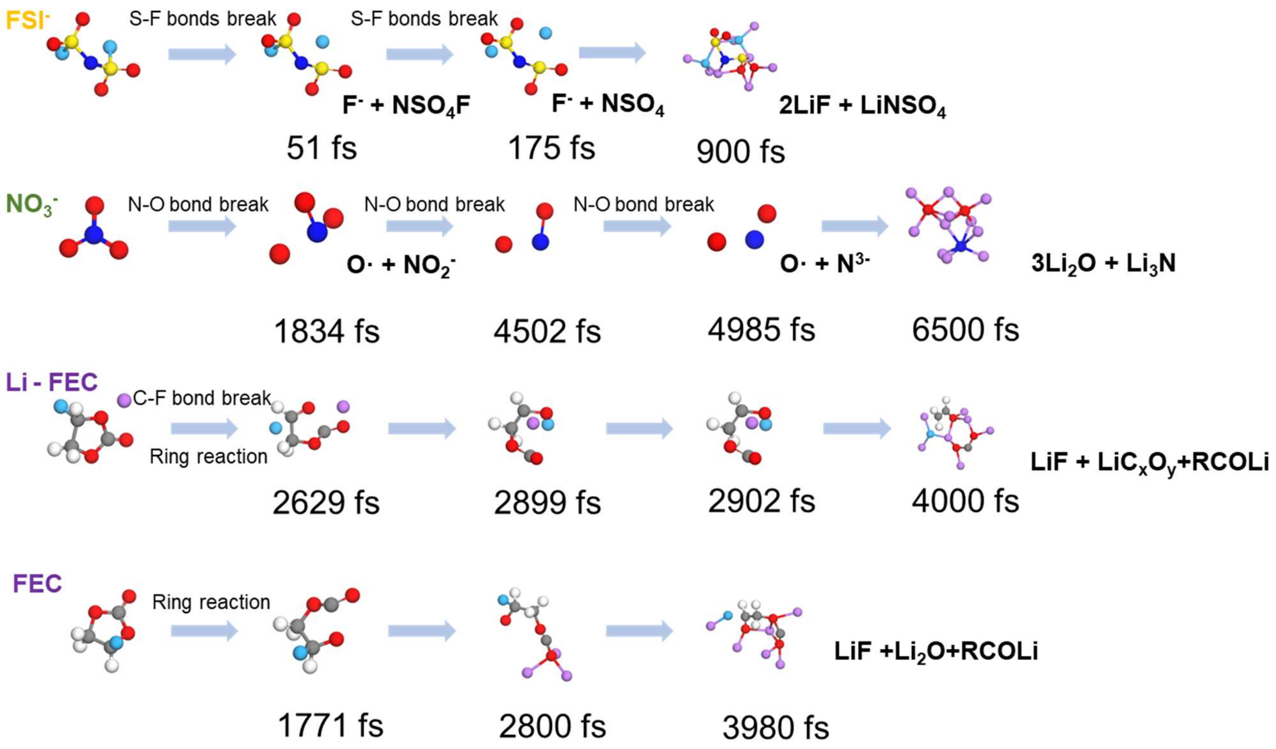

Figure S39. Evolution of different molecular reaction results from AIMD.

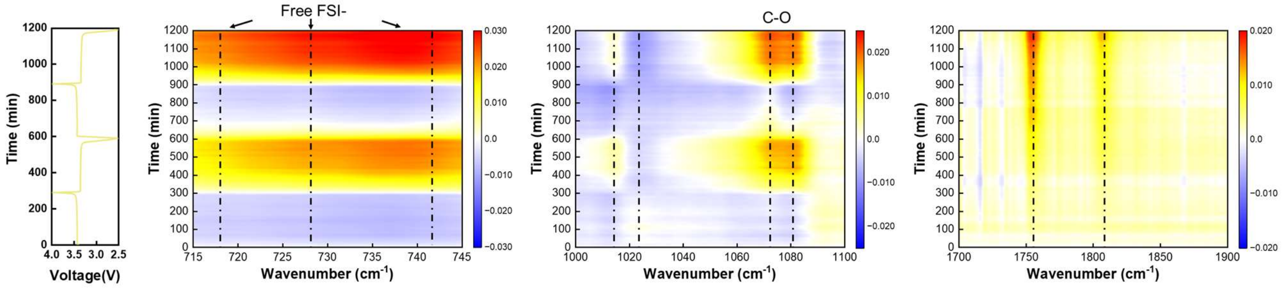

Figure S40. Voltage-time curves and corresponding contour map of the FTIR spectra during the charge/discharge process when using LCE

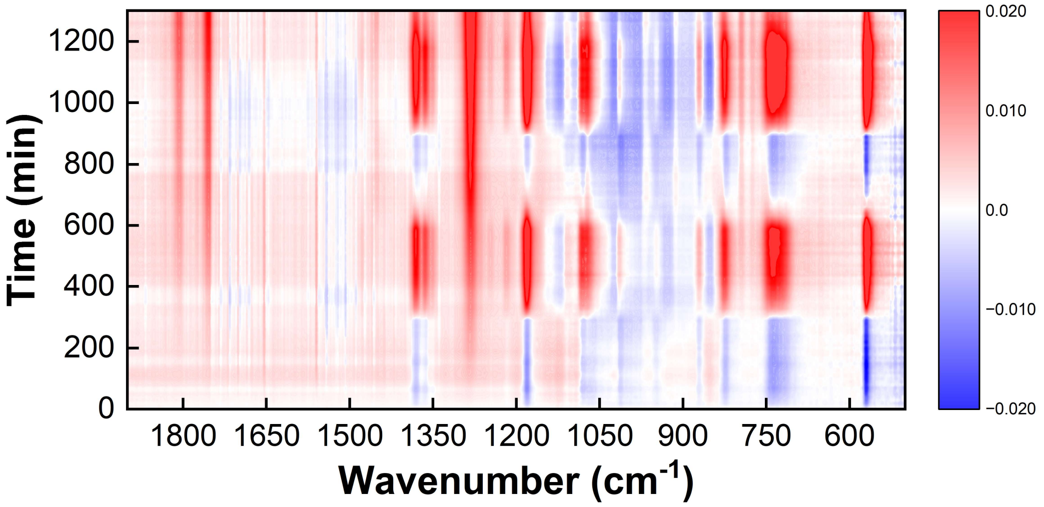

Figure S41. In situ FTIR of LFP||Li half cell with LCE (total)

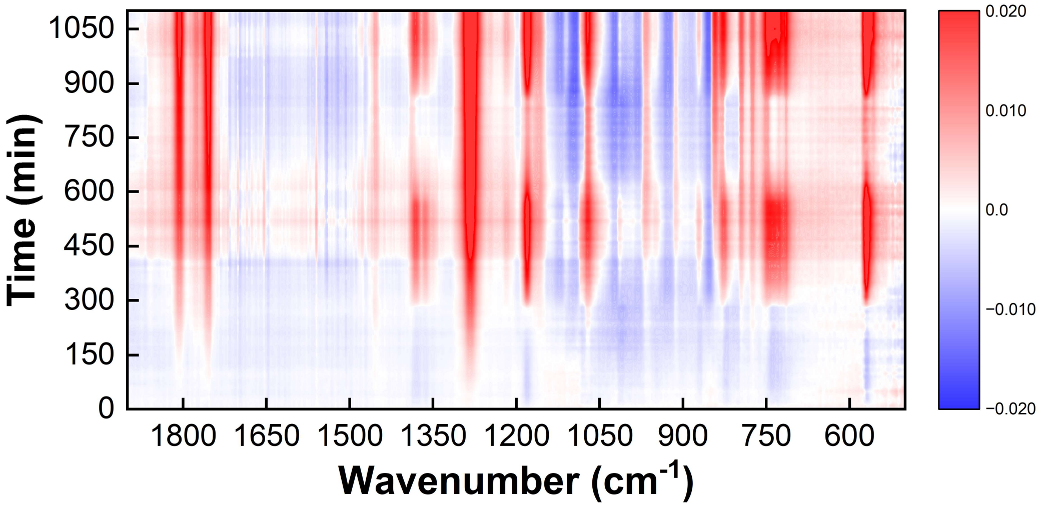

Figure S42. In situ FTIR of LFP||Li half cell with DN

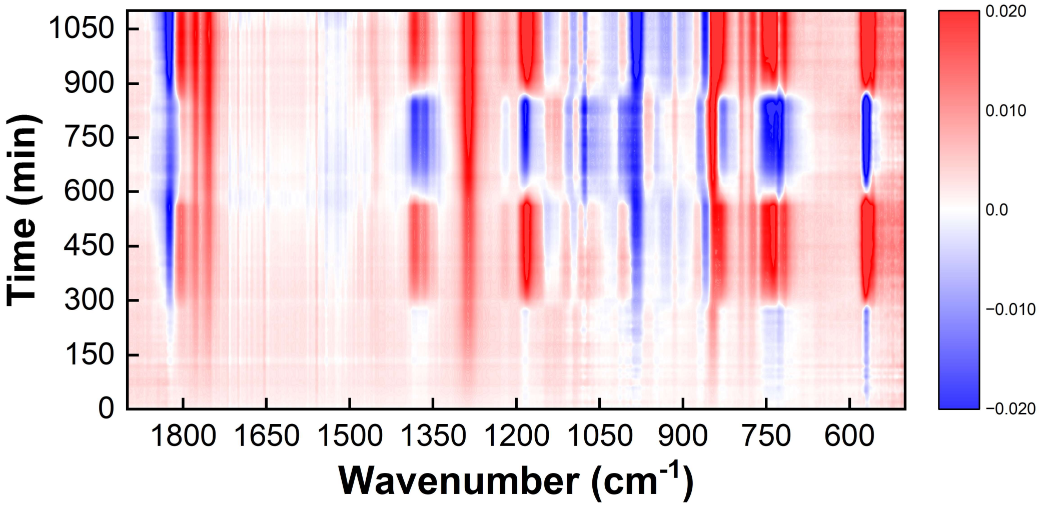

Figure S43. In situ FTIR of LFP||Li half cell with DFN

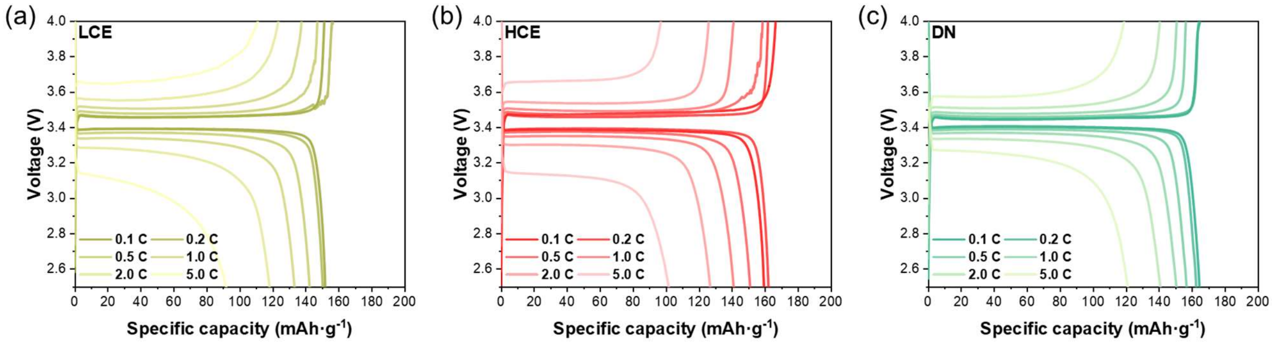

Figure S44. Charge-discharge curves of LFP||Li half-cells at different rate in (a) LCE, (b) HCE, and (c) DN.

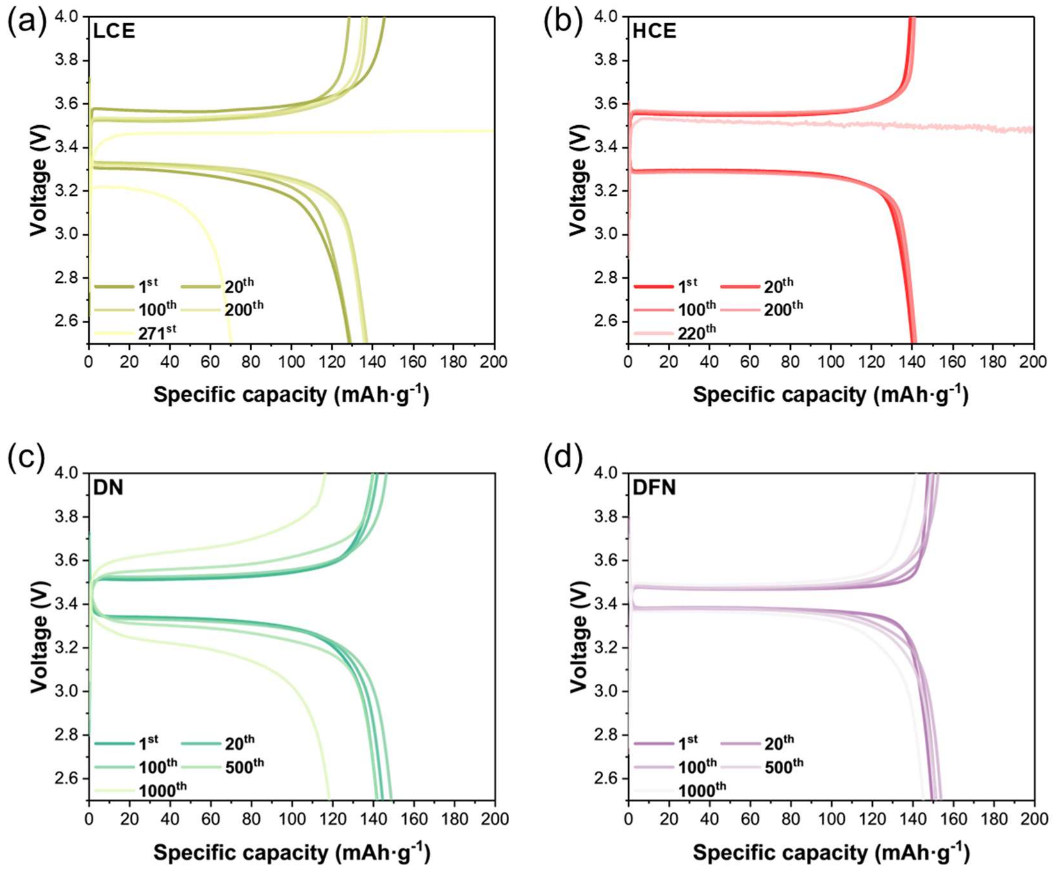

Figure S45. Charge-discharge curves of LFP||Li half-cells at different cycles in (a) LCE, (b) HCE, (c) DN, and (d) DFN.

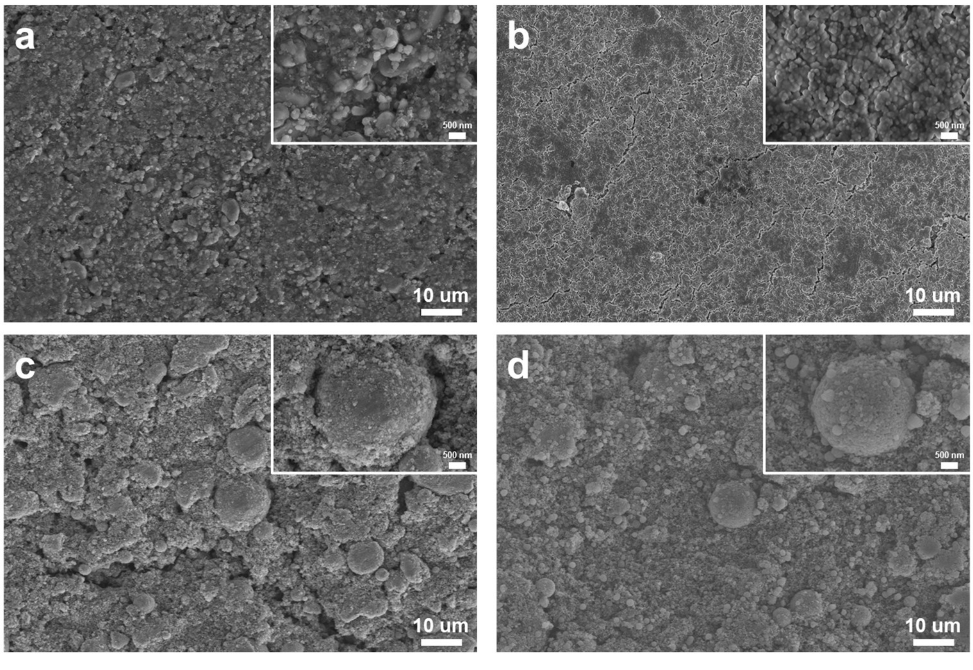

Figure S46. SEM images for the surface of LFP electrode after the 100 cycles at rate of 1.0 C in (a) LCE, (b) HCE, (c) DN, and (d) DFN.

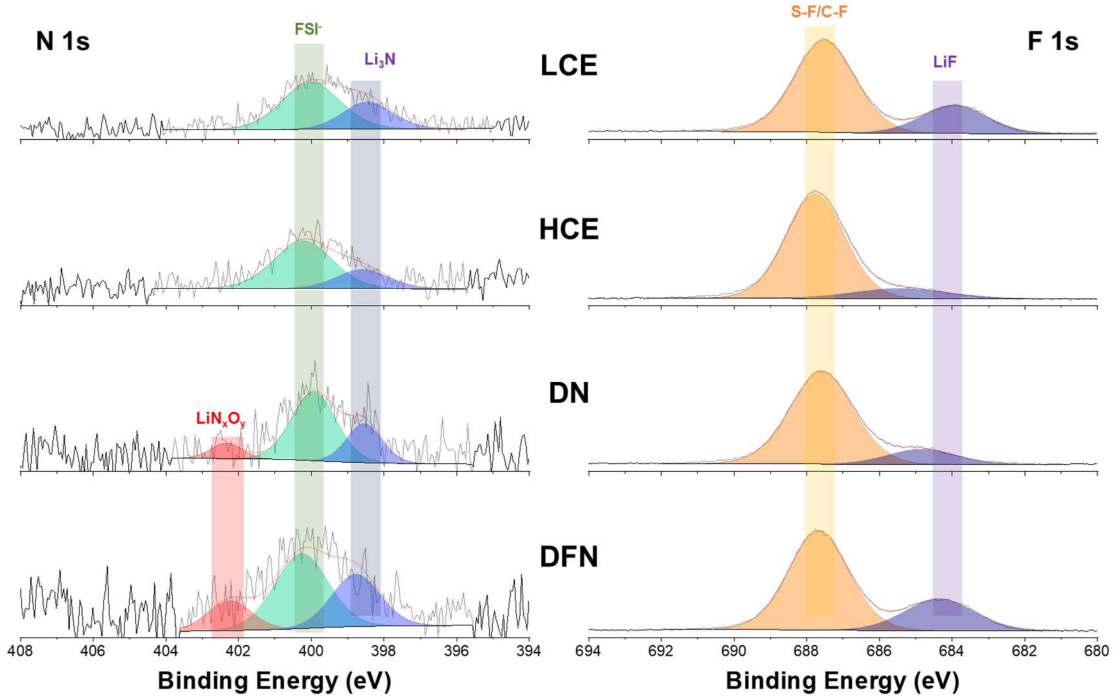

Figure S47. XPS of LFP cathode with different electrolytes

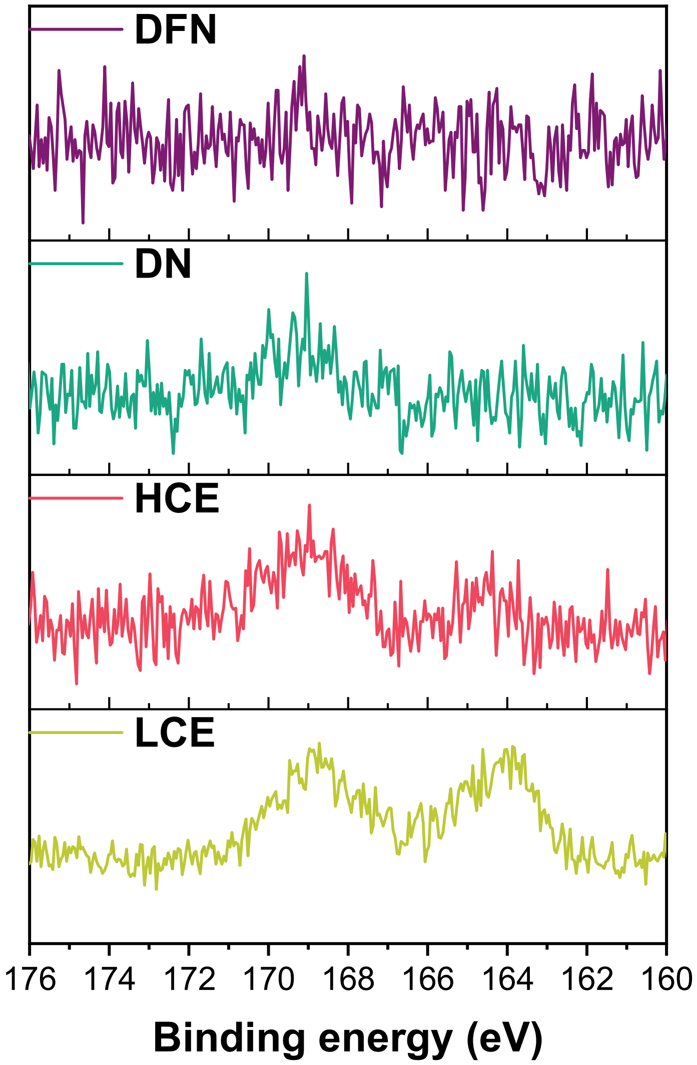

Figure S48. S 2p XPS of LFP cathode with different electrolytes

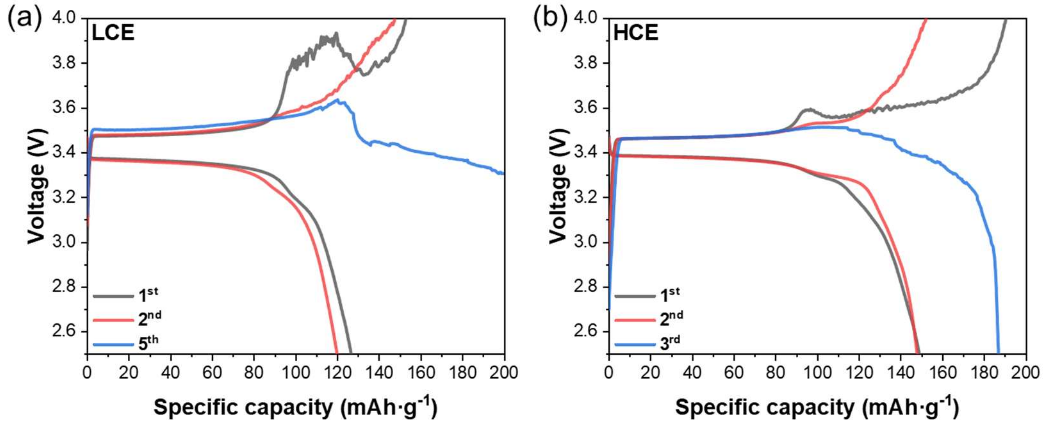

Figure S49. Different cycle's charge-discharge curves of LFP||Li half-cells in LCE and HCE electrolyte at 80 °C.

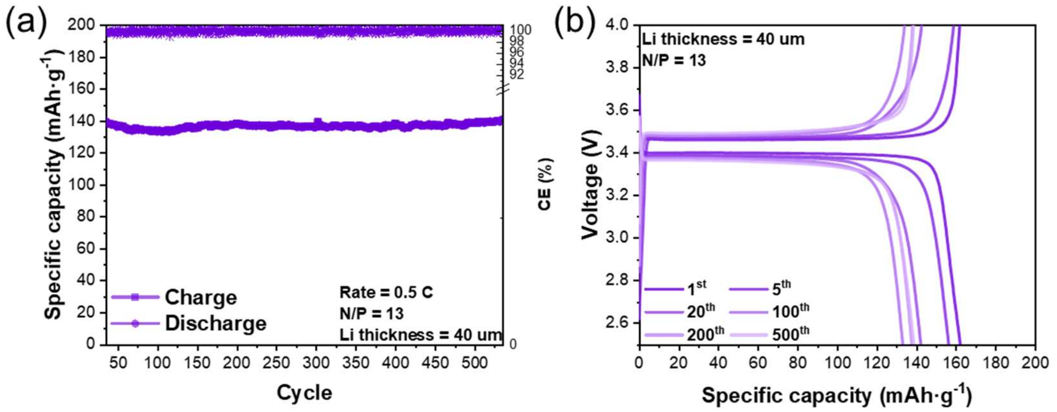

Figure S50. (a) Long-term cycling and (b) charge-discharge curves of LFP||Li (40 μm) full-cells at a rate of 0.5 C with DFN at room temperature.

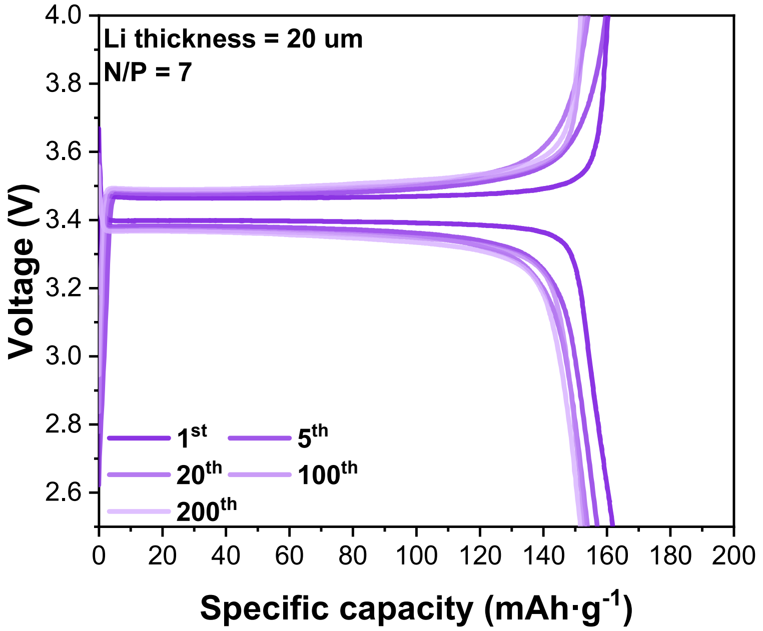

Figure S51. Charge-discharge curves of LFP||Li (20 μm) full-cells at a rate of 0.5 C with DFN at room temperature.

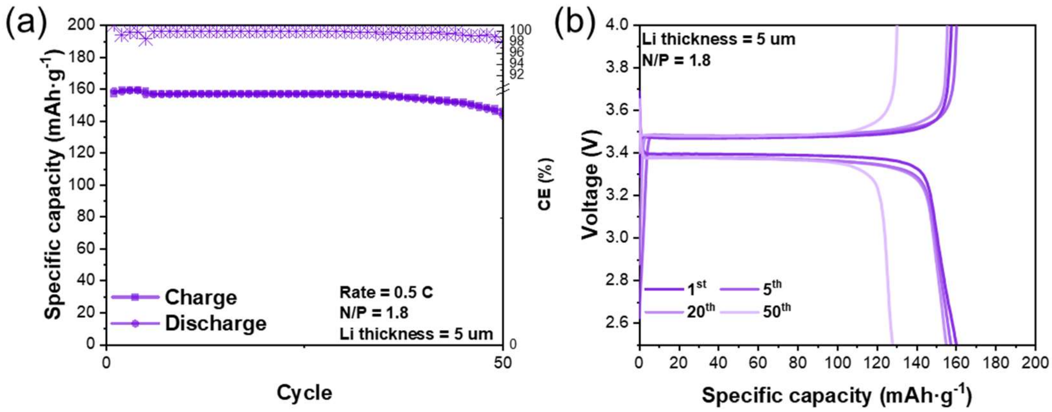

Figure S52. (a) Long-term cycling and (b) charge-discharge curves of LFP||Li (5 μm) full-cells at a rate of 0.5 C with DFN at room temperature.

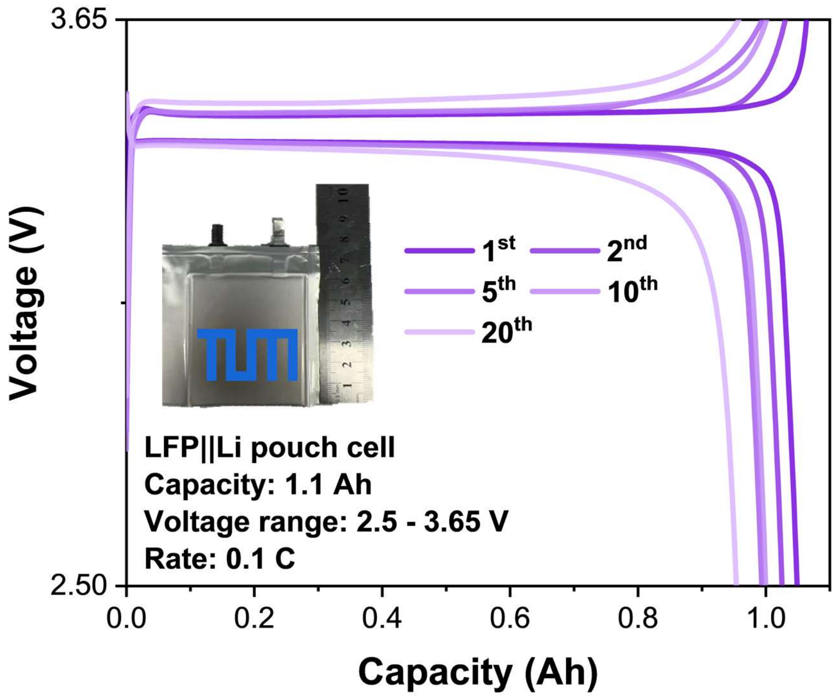

Figure S53. Charge-discharge curves of LFP||Li pouch full cells at a rate of 0.1 C with DFN at room temperature.

Supporting Tables

Adsorption structure			
**Li^+ - DOL**	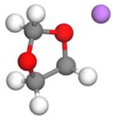	**Li^+ - G2 3**	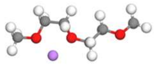
**Li^+ - DEE**	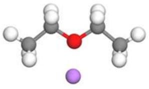	**Li^+ - G2 4**	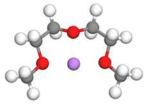
**Li^+ - THF**	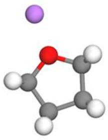	**Li^+ - EC**	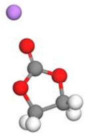
**Li^+ - DME 1**	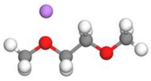	**Li^+ - FEC**	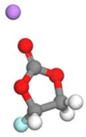
**Li^+ - DME 2**	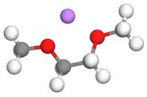	**Li^+ - FSI^-**	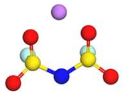
**Li^+ - G2 1**	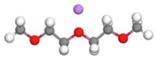	**Li^+ - NO_3^-**	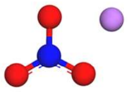
**Li^+ - G2 2**	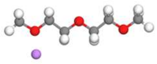		

Table S1. Adsorption structure between Li^+, solvents and anions

Sample	A (OCH_3)	B (CH_3OCH_2)	C ($CH_3OCH_2CH_2$)	D (Midvalue of B and C)	C-A value
Pure G2	3.26 ppm	3.44 ppm	3.51 ppm	3.49 ppm	0.25 ppm
1.0M LiFSI in G2	3.20 ppm	3.38 ppm	3.46 ppm	3.42 ppm	0.26 ppm
1.5M LiFSI in G2	3.16 ppm	3.34 ppm	3.44 ppm	3.38 ppm	0.28 ppm
2.0M LiFSI in G2	3.15 ppm	3.34 ppm	3.43 ppm	3.375 ppm	0.28 ppm
1.5M LiFSI in G2/EFC	3.06 ppm	3.27 ppm	3.38 ppm	3.32 ppm	0.32 ppm
DN	3.18 ppm	3.38 ppm	3.48 ppm	3.42 ppm	0.30 ppm
DFN	3.06 ppm	3.28 ppm	3.38 ppm	3.33 ppm	0.32 ppm

Table S2. ^{1}H Chemical shift details of different electrolytes and solvent.

Sample	CH_2/CH_3 Peak area ratio (A:B:C)
Pure G2	1.00 : 0.94 : 1.44
1.0M LiFSI in G2	1.00 : 0.98 : 1.50
1.5M LiFSI in G2	1.01 : 0.99 : 1.49
2.0M LiFSI in G2	1.00 : 1.01 : 1.54
1.5M FSI in G2/EFC	1.00 : 1.00 : 1.46
DN	1.00 : 0.99 : 1.50
DFN	1.00 : 1.01 : 1.50

Table S3. ^{1}H Chemical shift details of peak area.

Electrolyte	Battery (Type)	Cycle number (Temperature/rate)	Capacity retention (%)	Ref.
2.0 M LiFSI/0.2 M $LiNO_3$ in FEC/G4 (3:7, by volume)	Li||LFP (coin cell)	100 (90 °C) @1C<br>500 (25 °C) @1C	91<br>80	[7]
LiFSI: LiNO3: G4 (1:1:2.3 by mol)	Li||LFP (coin cell)	120 (90 °C) @0.2C<br>150 (25 °C) @0.1C	95<br>98	[8]
1.0 M $LiNO_3$/0.2 M LiODFB/0.05M $LiPF_6$ in G2	Li||LCO (coin cell)	400 (80 °C) @2C	80	[9]
1.0 M LiTFSI/0.5M $LiNO_3$ in TEP	Li||LFP (coin cell)	50 (75 °C) @1C<br>50 (100 °C) @1C	87<br>80	[10]
1M LiFSI/0.3 M $LiNO_3$ in FEC/GBL (1:2 v/v)	Li||NMC622 (coin cell)	100 (25 °C) @0.5C	80	[11]
1M $LiPF_6$/0.2 M $LiNO_3$ in EC: GTA: DMC=6: 1: 3, by volume	Li||NMC811 (coin cell)	180 (25 °C) @0.5C	84	[12]
LiFSI: G2: $LiNO_3$ (1:8:2 by mol)	Li||NMC811 (coin cell)	140 (25 °C) @0.5C<br>125 (25 °C) @0.2C	80<br>80	[13]
0.5 M $LiNO_3$/1 M LiDFOB in DME + 5%VC	Li||NMC811 (coin cell)	300 (25 °C) @0.66C	62	[14]
**1.0 M LiFSI/0.25 M $LiNO_3$ in G2/FEC (1:1 v/v)**	**Li||LFP (coin cell)**	**500 (80 °C) @1C**<br>**2000 (25 °C) @1C**	**96**<br>**90**	**This work**
	**Li (20 um)||LFP (coin cell)**	**200 (25 °C) @0.5C**	**100**	
	**Li (5 um)||LFP (coin cell)**	**50 (25 °C) @0.5C**	**91.7**	
	**Li||LFP (pouch cell, 1.1 Ah,)**	**20 (25 °C) @0.1C**	**91**	

Table S4. Performance comparison with previous literature related component on LMBs

References:

[1] a) J. Alvarado, M. A. Schroeder, T. P. Pollard, X. F. Wang, J. Z. Lee, M. H. Zhang, T. Wynn, M. Ding, O. Borodin, Y. S. Meng, K. Xu, Energy & Environmental Science **2019**, 12, 780; b) Y. Yang, Y. Yin, D. M. Davies, M. Zhang, M. Mayer, Y. Zhang, E. S. Sablina, S. Wang, J. Z. Lee, O. Borodin, C. S. Rustomji, Y. S. Meng, Energy & Environmental Science **2020**, 13, 2209.

[2] P. Liu, J. A. Rodriguez, Journal of the American Chemical Society **2005**, 127, 14871.

[3] a) T. Zheng, J. Xiong, X. Shi, B. Zhu, Y.-J. Cheng, H. Zhao, Y. Xia, Energy Storage Materials **2021**, 38, 599; b) Z. C. Wang, Y. Y. Sun, Y. Y. Mao, F. R. Zhang, L. Zheng, D. S. Fu, Y. B. Shen, J. C. Hu, H. L. Dong, J. J. Xu, X. D. Wu, Energy Storage Materials **2020**, 30, 228.

[4] a) M. J. Hossain, Q. Wu, E. J. Marin Bernardez, C. D. Quilty, A. C. Marschilok, E. S. Takeuchi, D. C. Bock, K. J. Takeuchi, Y. Qi, The Journal of Physical Chemistry Letters **2023**, 14, 7718; b) Q. Wu, M. T. McDowell, Y. Qi, Journal of the American Chemical Society **2023**, 145, 2473.

[5] a) T. Zheng, J. Xiong, B. Zhu, X. Shi, Y.-J. Cheng, H. Zhao, Y. Xia, Journal of Materials Chemistry A **2021**, 9, 9307; b) X. Q. Zhang, X. Chen, X. B. Cheng, B. Q. Li, X. Shen, C. Yan, J. Q. Huang, Q. Zhang, Angew Chem Int Ed Engl **2018**, 57, 5301.

[6] I. Hussain, S. Ullah, A. A. Khan, R. Ahmad, I. Ahmad, Computational Condensed Matter **2024**, 39, e00898.

[7] L.-P. Hou, X.-Q. Zhang, B.-Q. Li, Q. Zhang, Angewandte Chemie International Edition **2020**, 59, 15109.

[8] T. Chen, Z. Jin, Y. Liu, X. Zhang, H. Wu, M. Li, W. Feng, Q. Zhang, C. Wang, Angewandte Chemie International Edition **2022**, 61, e202207645.

[9] S. Yuan, S. Cao, X. Chen, J. Wei, Z. Lv, H. Xia, L. Chen, R. B. F. Ng, F. L. Tan, H. Li, X. J. Loh, S. Li, X. Feng, X. Chen, Journal of the American Chemical Society **2025**, 147, 4089.

[10] C. Ma, C. Fu, S. Chang, X. Xu, G. Zhang, Z. Liu, H. Huo, L. Fan, G. Yin, Y. Ma, ACS Energy Letters **2025**, 10, 1700.

[11] Y. Jie, X. Liu, Z. Lei, S. Wang, Y. Chen, F. Huang, R. Cao, G. Zhang, S. Jiao, Angewandte Chemie **2020**, 132, 3533.

[12] Z. Jin, Y. Liu, H. Xu, T. Chen, C. Wang, Angewandte Chemie **2024**, 136, e202318197.

[13] J. You, Q. Wang, R. Wei, L. Deng, Y. Hu, L. Niu, J. Wang, X. Zheng, J. Li, Y. Zhou, Nano-Micro Letters **2024**, 16, 257.

[14] Z. Jiang, T. Yang, C. Li, J. Zou, H. Yang, Q. Zhang, Y. Li, Advanced Functional Materials **2023**, 33, 2306868.
